# Buffering Effects of Physical Activity on Negative Life Events–Life Satisfaction Dynamics

**DOI:** 10.1002/smi.70198

**Published:** 2026-07-01

**Authors:** Daniel Groß, Jasmin Haffa, Wenke Möhring

**Affiliations:** ^1^ University of Education Schwäbisch Gmünd Schwäbisch Gmünd Germany; ^2^ Zentrum für Psychiatrie Reichenau Reichenau Germany

**Keywords:** buffering effects, life satisfaction, negative life events, physical activity

## Abstract

This study investigated whether physical activity buffers the association between negative life events and life satisfaction, distinguishing between dependent (e.g., divorce) and independent (e.g., bereavement) stressors as well as within‐ and between‐person processes across one‐ and 2‐year intervals (indicating short‐ and medium‐term intervals, respectively). Data from two large panel studies (*N* = 98,224) were analysed. Participants were classified as high (weekly or more) versus low (less than weekly) in physical activity. Three‐variable multiple‐group random intercept cross‐lagged panel models separated within‐person dynamics from between‐person associations. Dependent events predicted declines in life satisfaction across both datasets. This effect was attenuated among physically active individuals over 2‐year intervals but not over 1‐year intervals. Independent events showed no consistent medium‐term effects on life satisfaction. However, in the 1‐year interval, independent events were associated with subsequent declines in life satisfaction only among physically active individuals, whereas no association emerged among those with low physical activity. With respect to the reversed pathways, life satisfaction predicted subsequent dependent events across both groups and intervals. In contrast, higher life satisfaction was associated with a lower likelihood of subsequent independent events only among active individuals. At the between‐person level, buffering effects emerged only for the 2‐year association between independent events and life satisfaction. Physical activity is not a uniform buffering factor but relates to stress–well‐being dynamics in a manner that depends on stressor type, temporal scale, and analysis level. Effects are conditional and context‐specific rather than broadly protective.

## 1 Introduction

Negative life events—such as job loss, divorce, or serious illness—are common experiences across the life course and can substantially undermine individuals' life satisfaction (Luhmann et al. 2012, 2021). Life satisfaction represents the cognitive component of subjective well‐being and reflects individuals' global evaluations of their lives (Diener et al. 1999). Although negative life events are typically associated with declines in life satisfaction, individuals differ markedly in the magnitude and duration of these effects, indicating substantial heterogeneity in psychological adjustment and resilience (Kritzler et al. 2022). This variability may relate to exposure to specific stressors, which are typically distinguished between dependent and independent life events. Dependent life events are partly shaped by an individual's behaviour, decision, and interpersonal processes (e.g., divorce, job loss), whereas independent life events occur largely outside personal control (e.g., bereavement; Liu et al. 2024; Rnic et al. 2023). Differentiating between these stressor types allows for a more precise understanding of individual differences, not only in stress reactivity but also in stress exposure, as vulnerability and resilience processes may operate differently depending on the degree of personal involvement when facing stressful events.

Although this distinction is widely used in longitudinal research (Rnic et al. 2023), it should be interpreted with caution. Perceived controllability exists on a continuum, and even ostensibly independent events may be experienced as more or less controllable depending on individuals' resources, prior experiences, and contextual factors. Likewise, events commonly classified as dependent may be perceived as largely uncontrollable by those affected. Because respondents' *subjective* appraisals of negative life events are not directly assessed in the panel data used in the present study (HILDA and SOEP), we rely on this categorisation as an analytical heuristic while acknowledging its inherent subjectivity and limitations.

Importantly, prior research suggests that dependent and independent life events differ systematically in how they affect well‐being over time. Independent negative life events, such as the loss of a loved one, typically lead to an immediate decline in life satisfaction followed by a gradual return to baseline levels over time (Asselmann and Specht 2023; Groß and Haffa 2026). This pattern aligns with set‐point theory, which posits that well‐being fluctuates around a person‐specific baseline and that the effects of positive and negative events tend to attenuate over time (Headey and Wearing 1989; Lykken and Tellegen 1996). In contrast, dependent negative life events, such as job loss, are less likely to show full adaptation and have been shown to induce longer‐lasting reductions in life satisfaction, indicating potential shifts in the individual set‐point (Groß and Haffa 2026; Lucas et al. 2004).

In addition to these adaptation processes, theoretical accounts of stress also emphasise reciprocal associations between well‐being and subsequent stress exposure. Stress generation theory proposes that initial lower well‐being and maladaptive coping processes may increase the likelihood of experiencing subsequent stressors. This seems particularly true for dependent negative life events that are partly shaped by individuals' own behaviours and interpersonal processes. However, it needs to be noted that this reversed pathway was mainly found in clinical and high‐risk populations (Hammen 1991; Liu et al. 2024; Rnic et al. 2023). Thus, life satisfaction may not only be an outcome of stressful experiences but may also prospectively influence individuals' exposure to future negative life events. Taken together, set‐point theory (Headey and Wearing 1989; Lykken and Tellegen 1996) and stress generation theory (Hammen 1991; Liu et al. 2024; Rnic et al. 2023) suggest that dependent and also independent negative life events may differ in their immediate effects on life satisfaction as well as in their longer‐term dynamic interplay with well‐being.

Against this background, the present study investigates whether physical activity serves as a resource that may buffer the longitudinal associations between dependent and independent negative life events and life satisfaction. Physical activity encompasses any bodily movement produced by skeletal muscles that requires energy expenditure, such as walking, cycling, sports participation, or structured exercise (Caspersen et al. 1985). The research question of physical activity as a stress‐buffering resource builds upon studies showing that regular engagement in physical activity has been linked to enhanced stress‐coping capacity (Gerber et al. 2025). Additionally, physical activity has consistently been associated with improved stress regulation, and higher subjective well‐being (Gerber and Pühse 2009; Klaperski 2018). Studies in everyday stress contexts—such as academic examinations—indicate that physically active individuals experience fewer adverse effects of stress on affect and well‐being (Flueckiger et al. 2016; Gerstberger et al. 2023; Puterman et al. 2017; von Haaren et al. 2015). Research using major life events as stress indicators points in a similar direction (Gerber and Pühse 2009; Klaperski 2018). Several mechanisms may explain why physical activity functions as a resilience‐promoting resource in the context of negative life events. It is associated with improved affective well‐being, reduced stress reactivity, greater self‐efficacy, and social integration, thereby supporting both emotional recovery and behavioural coping processes (Gerber and Pühse 2009; Klaperski 2018; Morava et al. 2024; Pauly et al. 2020; Prochnow and Patterson 2022; Rehder et al. 2026). However, most prior research has focused on psychobiological indicators (e.g., cortisol) or psychopathological outcomes such as depression (Harris et al. 2006; Heaney et al. 2014; O’Dougherty et al. 2012; Szuhany et al. 2023), rather than assessing life satisfaction as a dependent variable. Moreover, findings vary substantially in design, population, and temporal scope, limiting comparability across studies.

The present study builds directly on prior research by Groß and Haffa (2026) who analysed volunteering as a potential buffering resource, and used the same longitudinal datasets as in this present study—the Household, Income and Labour Dynamics in Australia (HILDA) Survey (1‐year intervals; Watson and Wooden 2012) and the German Socio‐Economic Panel (SOEP; 2‐year intervals; Goebel et al. 2019). Applying multiple‐group Random Intercept Cross‐Lagged Panel Models (RI‐CLPMs), Groß and Haffa (2026) found evidence that volunteering can serve as a buffering resource by explicitly distinguishing within‐person processes from stable between‐person associations. However, these effects varied according to stressor type, temporal scale, and analysis level, highlighting their conditional nature rather than a broadly protective role.

In RI‐CLPMs, associations between random intercepts reflect between‐person differences. Within‐person autoregressive and cross‐lagged effects capture deviations from individuals' typical levels over time (Mulder and Hamaker 2021). This distinction is crucial, as within‐person processes cannot be inferred from between‐person correlations. A negative lagged effect indicates that negative deviations from an individual's typical level in one variable are followed by positive deviations in another variable at the next measurement point, and vice versa. Conversely, a positive lagged effect indicates that positive deviations in one variable are followed by positive deviations in the other variable at the next time point, whereas negative deviations are followed by negative deviations in the subsequent wave (Mulder and Hamaker 2021). A more detailed explanation of model parameters is provided in the electronic supplementary material (ESM).

Although volunteering and physical activity are conceptually distinct behaviours, both may function as behavioural resilience resources that facilitate adaptation to stress through behavioural, psychological, and social processes (Nichol et al. 2024; Rahmati et al. 2024). The present study therefore extends recent findings on volunteering by examining whether physical activity moderates the reciprocal associations between negative life events and life satisfaction. This also includes the analysis whether physical activity moderates the reverse pathway—from life satisfaction to subsequent negative life events. Such a result would indicate that physical activity, potentially through enhanced affective well‐being, greater physiological resilience, and increased social integration, weakens this association and reduces stress generation.

## 2 Present Studies, Theoretical Framework and Hypotheses

Both present studies examine whether physical activity (a) buffers the detrimental effects of dependent and independent negative life events on life satisfaction and (b) attenuates the prospective association between low life satisfaction and subsequent negative life events. The study is structurally and methodologically identical to the study by Groß and Haffa (2026), and the hypotheses are derived accordingly.

Analyses use annual HILDA data (Study 1, Watson and Wooden 2012) and biennial SOEP data (Study 2, Goebel et al. 2019), applying RI‐CLPMs (Mulder and Hamaker 2021) to separate within‐person dynamics from between‐person differences across Australian and German contexts. Australia and Germany differ in physical activity patterns in the population. Guthold et al. (2018) reported that the prevalence of insufficient physical activity was 30.4% in Australia and 42.2% in Germany across both sexes combined. Insufficient physical activity was defined as not meeting WHO recommendations for health‐enhancing physical activity (i.e., at least 150 min of moderate‐intensity or 75 min of vigorous‐intensity activity per week, or an equivalent combination). These findings indicate that a larger proportion of the German population does not meet recommended activity levels. The two countries also differ slightly in average life satisfaction. According to the World Happiness Report 2024, Australia reported a higher average life satisfaction score (6.974; rank 11) than Germany (6.753; rank 22), suggesting somewhat higher subjective well‐being in Australia (Helliwell et al. 2025). While cultural differences are beyond the scope of the present study, these variations may affect comparability across the two datasets.

### 2.1 Theoretical Framework

Building on set‐point theory (Headey and Wearing 1989; Lykken and Tellegen 1996) and stress generation theory (Hammen 1991; Liu et al. 2024; Rnic et al. 2023), life satisfaction is conceptualised as a dynamic construct that fluctuates around relatively stable baseline levels while being reciprocally linked to negative life events. Set‐point theory assumes that individuals generally adapt to life events over time and return towards baseline levels after temporary deviations (Headey and Wearing 1989; Lykken and Tellegen 1996). Evidence suggests that this pattern is particularly evident following independent life events: although life satisfaction declines shortly after such events, it tends to gradually recover towards baseline levels over time (Asselmann and Specht 2023; Groß and Haffa 2026). In contrast, dependent negative life events (e.g., job loss, divorce) may also induce longer‐lasting shifts in baseline life satisfaction (Lucas et al. 2004), suggesting potential set‐point changes.

Stress generation theory (Hammen 1991; Liu et al. 2024; Rnic et al. 2023) complements this view by proposing that life satisfaction also predicts future stress exposure. That appears to apply particularly for dependent negative life events embedded in behavioural and interpersonal processes. Consequently, this may create a self‐reinforcing feedback loop between stress exposure and well‐being.

Within the RI‐CLPM, within‐person effects capture short‐term deviations from typical levels of an individuals, whereas correlations among the random intercept factors capture stable between‐person differences that approximate longer‐term equilibrium processes and are conceptually similar to cross‐sectional correlations, but are estimated using information from multiple measurement waves.

Physical activity is conceptualised as a stable behavioural resource reflecting habitual engagement in health‐promoting behaviour.

### 2.2 Hypothesis 1 (Dependent Life Events)

Physical activity is expected to attenuate the within‐person bidirectional association between dependent negative life events and life satisfaction, reducing both stress‐to‐well‐being and well‐being‐to‐stress effects. This buffering effect is expected to be more pronounced in the 2‐year interval, as regulatory and behavioural adaptations accumulate over time.

### 2.3 Hypothesis 2 (Independent Life Events)

Independent negative life events are expected to predict short‐term declines in life satisfaction in the 1‐year interval. Adaptation towards baseline levels is expected in the 2‐year interval across both groups. Thus, physical activity is expected to moderate the short‐term associations by accelerating adaptation processes, resulting in buffering effects in the 1‐year interval only. Higher physical activity is additionally expected to be associated with lower temporal stability (i.e., no significant autoregressive effect) of independent negative life events.

### 2.4 Hypothesis 3 (Between‐Person Effects)

Extending these within‐person processes, physical activity is expected to moderate the association between independent negative life events and life satisfaction in the 2‐year interval (SOEP), while no moderation is expected in the 1‐year interval (HILDA). This expectation is grounded in set‐point theory (Headey and Wearing 1989; Lykken and Tellegen 1996), which suggests that independent negative life events induce temporary deviations followed by adaptation. Since between‐person differences reflect cumulative, emergent patterns of stress reactivity which require sufficient temporal aggregation, these effects may not be fully observable in the 1‐year interval.

In contrast, no buffering effect is expected for dependent negative life events at the between‐person level. In line with stress generation theory (Hammen 1991; Liu et al. 2024; Rnic et al. 2023), dependent stressors emerge from stable person–environment transactions linking life satisfaction, coping, and interpersonal processes in a self‐reinforcing feedback system. As a result, within‐person buffering effects of physical activity are not expected to translate into attenuation of stable between‐person associations.

## 3 General Method

### 3.1 Samples, Procedure and Measures

We tested the buffering effect of physical activity using two large‐scale longitudinal panel studies with a combined sample of 98,224 individuals: HILDA Survey (Watson and Wooden 2012; Study 1) and the SOEP (Goebel et al. 2019; Study 2). Both panel studies assessed dependent and independent negative life events as well as life satisfaction in large adult samples. In both panels, participants were recruited through probability‐based household sampling designs aimed at achieving population representativeness at baseline. Participants received incentives to take part.

Using data from the HILDA panel (21 annual waves spanning 2002–2022), buffering effects were examined over 1‐year intervals. In HILDA, physical activity was operationalised as self‐reported moderate‐to‐vigorous physical activity. Using data from the SOEP (8 waves spanning 2007–2021), buffering effects were examined over 2‐year intervals, as sport participation was assessed biennially from 2007 onward. In SOEP, physical activity was captured as sport participation in leisure time. Despite differences in operationalisation and response formats, both measures of physical activity reflected regular engagement in physical activity. To differentiate between individuals with generally higher versus lower levels of physical activity, we calculated each individual's median level of physical activity across all available waves. Based on this median, participants were categorised into two groups: low physical activity (less than once per week) and high physical activity (at least once per week). This cutoff was chosen to enhance comparability between the two panels despite differences in measurement instruments. Although moderation effects can also be examined using time‐varying continuous moderators (e.g., Ozkok et al. 2022; Speyer et al. 2023), we opted for a time‐invariant categorical grouping. This decision was motivated by the high computational demands of incorporating a continuous time‐varying moderator in large panel datasets, differences in response formats across panels, and the aim of ensuring methodological comparability with Groß and Haffa (2026); see also Falkenström (2024). We categorised negative life events a priori into two groups in both panels (see ESM): independent events, which occur more likely outside an individual's influence and are typically beyond their control, and dependent events, which are considered as being partially influenced by an individual characteristics or behaviour and are generally perceived as more controllable. This classification aligns with the categorisation used by Maciejewski et al. (2021). Life satisfaction was measured using a global single‐item indicator assessing participants' overall evaluation of their lives.

### 3.2 Statistical Analysis

Analyses were conducted using RI‐CLPMs (Mulder and Hamaker 2021). To test the buffering role of physical activity, we estimated three‐variable multiple‐group RI‐CLPMs including independent negative life events, dependent negative life events, and life satisfaction. Details on model constraints, model comparisons, and handling of missing data are provided in the ESM.

### 3.3 Estimation and Model Evaluation

All analyses were conducted in R (Version 4.4.0). Because multivariate normality assumptions were violated in both panel datasets, models were estimated using robust maximum likelihood estimation (MLR; Lai 2018). Model fit was evaluated using conventional cutoff criteria: RMSEA < 0.06, SRMR ≤ 0.08, and CFI ≥ 0.95 (Schreiber et al. 2006). Cross‐lagged effect sizes were interpreted using standardized estimates, with values of 0.03, 0.07, and 0.12 indicating small, moderate, and large effects, respectively (Orth et al. 2024). For correlations between random intercept factors, which are comparable to cross‐sectional correlations based on multiwave data, effect sizes of 0.10, 0.30, and 0.50 were interpreted as small, moderate, and large, respectively (Cohen 1992). For clarity, we report standardized effects. However, standardized coefficients may vary across timepoints due to differences in the residual variances of predictors and outcomes, even when unstandardised paths are constrained (Mulder 2023). When standardized estimates varied across waves, we indicated the range of variation using brackets (e.g., $\beta_{DE\_NLE∼LIFE}$ = [–0.027, −0.037]). The analyses were not preregistered. All analysis code is available on the Open Science Framework (OSF) at: https://osf.io/qyb34.

## 4 Study 1: The Household, Income and Labour Dynamics in Australia (HILDA)

### 4.1 Method

We analysed 21 waves of the HILDA Survey (2002–2022). HILDA, initiated in 2001, is a nationally representative, annual household survey capturing economic and personal well‐being, labour market outcomes, and family dynamics (Watson and Wooden 2012). Wave 2001 was excluded because the life events checklist was first introduced in 2002. The analytical sample included 7669 low physically active and 23,608 high physically active individuals. Descriptive statistics for age, gender, independent and dependent negative life events, and life satisfaction across waves and activity groups are reported in Supporting Information S1: Table 1 of the ESM. Detailed descriptions and operationalizations of all variables are provided in the ESM.

### 4.2 Results

**FIGURE 1:**
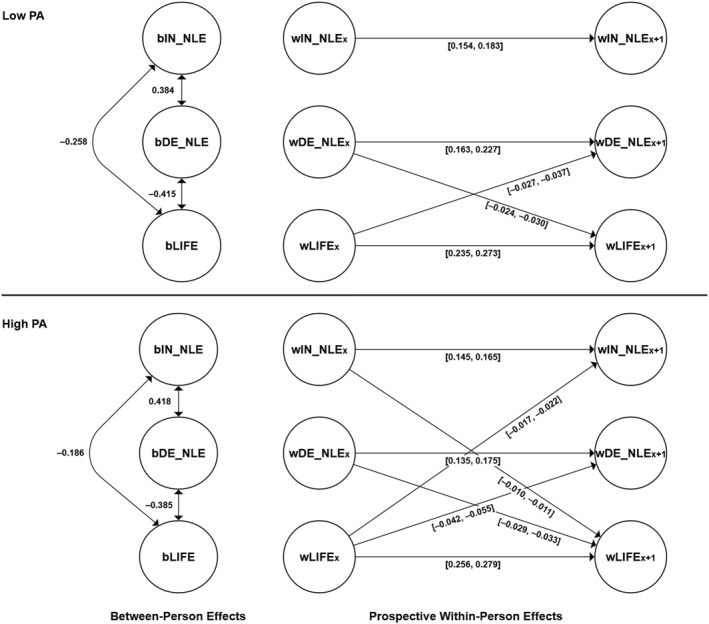
Results for the Multiple‐Group Random Intercept Cross‐Lagged Panel Model in Study 1 (HILDA). Only significant effects are shown. The prefix b denotes between‐person effects. The prefix w denotes within‐person effects. DE_NLE, dependent negative life events; IN_NLE, independent negative life events; LIFE, life satisfaction; PA, moderate‐to‐vigorous physical activity. Standardized estimates are reported; [ ], describes the values between which the standardized effects vary.

The results of the multiple‐group comparison indicated that some of the time‐invariant autoregressive and cross‐lagged parameters differed between the low and high physically active group. It can therefore be assumed that there are group‐specific effects. Supporting Information S1: Table 2 in the ESM presents a complete summary of the results, including the RI‐CLPM model parameter estimates, which all showed good fit. Additionally, all significant between‐person random intercept correlations, autoregressive effects and lagged effects are visually represented in Figure 1.

#### 4.2.1 Random Intercept Correlations (Between‐Person)

For both groups, low physical activity ($T_{DE\_NLE∼∼LIFE}=–0.415,T_{IN\_NLE∼∼LIFE}=–0.258,T_{IN\_NLE∼∼DE\_NLE}=0.384)$ and high physical activity ($T_{DE\_NLE∼∼LIFE}=–0.385,T_{IN\_NLE∼∼LIFE}=–0.186,T_{IN\_NLE∼∼DE\_NLE}=0.418)$, we found moderate‐to‐large negative correlations between the random intercepts of dependent negative life events and life satisfaction, small‐to‐moderate negative correlations between the random intercepts of independent negative life events and life satisfaction, and moderate‐to‐large negative correlations between the random intercepts of independent negative life events and dependent negative life events.

#### 4.2.2 Autoregressive Effects (Within‐Person)

We found significant positive autoregressive effects for all variables in the low physical activity group ($\gamma_{IN\_NLE∼IN\_NLE}=\left[0.154,0.183\right],\gamma_{DE\_NLE∼DE\_NLE}=\left[0.163,0.227\right],\gamma_{LIFE∼LIFE}=\left[0.234,0.273\right]$) and in the high physical activity group ($\gamma_{IN\_NLE∼IN\_NLE}=\left[0.145,0.165\right],\gamma_{DE\_NLE∼DE\_NLE}=\left[0.135,0.175\right],\gamma_{LIFE∼LIFE}=\left[0.256,0.279\right]$).

#### 4.2.3 Lagged Effects (Within‐Person)

We found small negative bidirectional effects between dependent negative life events and life satisfaction in the low physical activity group ($\beta_{DE\_NLE∼LIFE}$ = [–0.027, −0.037]; $\beta_{LIFE∼DE\_NLE}$ = [–0.024, −0.030]) and in the high physical activity group ($\beta_{DE\_NLE∼LIFE}$ = [–0.042, −0.055]; $\beta_{LIFE∼DE\_NLE}$ = [–0.029, −0.033]). In the high physical activity group, we also found small negative bidirectional effects between independent negative life events and life satisfaction ($\beta_{IN\_NLE∼LIFE}$ = [–0.017, −0.022]; $\beta_{LIFE∼IN\_NLE}$ = [–0.010, −0.011]), whereas there were no significant effects for the low physical activity group.

### 4.3 Summary of the Results

At the within‐person level, deviations above an individual's typical level of dependent negative life events (i.e., experiencing more dependent life events as usual) were associated with subsequent declines in life satisfaction. Deviations below the typical level (i.e., experiencing fewer dependent life events as usual) were associated with subsequent increases in life satisfaction. Conversely, lower‐than‐usual life satisfaction predicted an increased likelihood of experiencing dependent negative life events, whereas higher‐than‐usual life satisfaction predicted a reduced likelihood of such events. Therefore, dependent life events and life satisfaction are reciprocally related to one another, which holds true regardless of varying levels of physical activity.

A different pattern emerged for independent negative life events. Among individuals with low levels of physical activity, no systematic within‐person coupling between independent negative life events and life satisfaction was observed, indicating that deviations in one construct did not predict changes in the other over time. In contrast, among physically active individuals, deviations above an individual's typical level of independent negative life events predicted subsequent declines in life satisfaction and vice versa. Moreover, lower‐than‐usual life satisfaction predicted a higher likelihood of experiencing independent negative life events, whereas higher‐than‐usual life satisfaction predicted a reduced likelihood of such events. This bidirectional association suggests that the dynamic interplay between largely uncontrollable stressors and well‐being depends on engagement in physical activity, with stronger within‐person coupling among active individuals. No differences emerged between physical activity groups with respect to autoregressive effects, indicating comparable within‐person stability of life satisfaction and negative life events across groups. Similarly, at the between‐person level, physical activity groups did not differ. Dependent and independent negative life events were positively correlated, and both were negatively associated with life satisfaction, with stronger associations observed for dependent events.

## 5 Study 2: German Socio‐Economic Panel (SOEP)

### 5.1 Method

We analysed eight biennial waves of the SOEP (Goebel et al. 2019) from 2007 to 2021. The SOEP is a nationally representative, annual longitudinal study of private households and individuals, administered by the German Institute for Economic Research (DIW Berlin) since 1984. The 2007 wave marked the first inclusion of the negative life event death of a child, which is why our analysis begins in that year. Although SOEP data are collected annually, leisure‐time sport participation is assessed every two years; therefore, we utilised eight waves covering 2007–2021. The analytical sample comprised 40,818 individuals classified as low physically active and 26,129 as high physically active. Descriptive statistics for age, gender, independent and dependent negative life events, and life satisfaction across waves and activity groups are reported in Supporting Information S1: Table 3 of the ESM. Detailed descriptions and operationalizations of all variables are provided in the ESM.

### 5.2 Results

**FIGURE 2:**
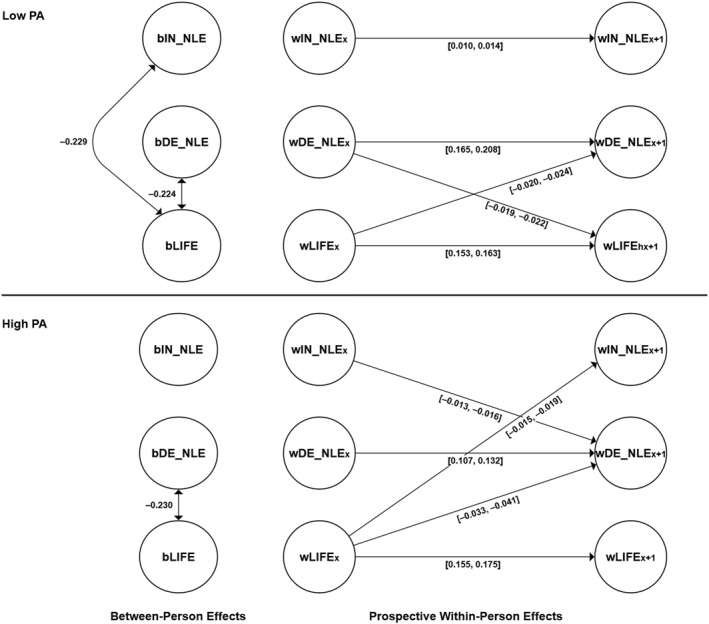
Results for the Multiple‐Group Random Intercept Cross‐Lagged Panel Model in Study 2 (SOEP). Only significant effects are shown. The prefix b denotes between‐person effects. The prefix w denotes within‐person effects. DE_NLE, dependent negative life events; IN_NLE, independent negative life events; LIFE, life satisfaction; PA, sport participation in leisure time. Standardized estimates are reported; [ ], describes the values between which the standardized effects vary.

The results of the multiple‐group comparison indicated that some of the time‐invariant autoregressive and cross‐lagged parameters differ between the low and high physical active group. It can therefore be assumed that there are group‐specific effects. Supporting Information S1: Table 4 in the ESM presents a complete summary of the results, including the RI‐CLPM model parameter estimates, which all showed good fit. Additionally, all significant between‐person random intercept correlations, autoregressive effects and lagged effects are visually represented in Figure 2.

#### 5.2.1 Random Intercept Correlations (Between‐Person)

For the low physical activity ($T_{DE\_NLE∼∼LIFE}=–0.224)$ and the high physical activity group ($T_{DE\_NLE∼∼LIFE}=–0.230)$, we found small‐to‐moderate negative correlations between the random intercepts of dependent negative life events and life satisfaction. In the low physical activity group, but not in the high physical activity group, we also found a negative small‐to‐moderate correlation between the random intercepts of independent negative life events and life satisfaction ($T_{DE\_NLE∼∼LIFE}=–0.229)$.

#### 5.2.2 Autoregressive Effects (Within‐Person)

We found significant positive autoregressive effects for all variables in the low physical activity group ($\gamma_{IN\_NLE∼IN\_NLE}=\left[0.010,0.014\right],\gamma_{DE\_NLE∼DE\_NLE}=\left[0.165,0.208\right],\gamma_{LIFE∼LIFE}=\left[0.153,0.163\right]$). In the high physical activity group, we found significant positive autoregressive effects for dependent negative life events and life satisfaction ($\gamma_{DE\_NLE∼DE\_NLE}=\left[0.107,0.132\right],\gamma_{LIFE∼LIFE}=\left[0.155,0.175\right]$), but not for independent life events.

#### 5.2.3 Lagged Effects (Within‐Person)

We found small negative bidirectional effects between dependent negative life events and life satisfaction ($\beta_{DE\_NLE∼LIFE}$ = [–0.020, −0.024]; $\beta_{LIFE∼DE\_NLE}$ = [–0.019, −0.022]) in the low physical activity group. Bidirectional effects between independent negative life events and life satisfaction were not significant in the low physical activity group but in the high physical activity group. In high physical activity group, we found a small negative association of life satisfaction on both dependent negative life events ($\beta_{DE\_NLE∼LIFE}$ = [–0.033, −0.041]) and independent negative life events ($\beta_{DE\_NLE∼LIFE}$ = [–0.015, −0.019]). Additionally, we found a small negative association of independent negative life events on dependent negative life events ($\beta_{DE\_NLE∼IN\_NLE}$ = [–0.013, −0.016]) in the high physical activity group.

### 5.3 Summary of the Results

At the within‐person level, among individuals with low levels of physical activity, dependent negative life events and life satisfaction were reciprocally related: deviations above an individual's typical level of dependent stressors (i.e., experiencing more dependent life events as usual) predicted subsequent declines in life satisfaction, while deviations below the typical level (i.e., experiencing fewer dependent life events as usual) predicted increases in life satisfaction. For the reversed relationship, it was found that lower‐than‐usual life satisfaction increased the likelihood of future dependent stressors, and higher‐than‐usual life satisfaction decreased it. Importantly, among physically active individuals, this reciprocal association was partially attenuated: dependent negative life events no longer predicted subsequent declines in life satisfaction, although within‐person deviations in life satisfaction continued to systematically relate to subsequent dependent stressors. This asymmetry suggests that physical activity buffers the consequences of behaviour‐linked stressors without fully disrupting the mechanisms through which well‐being shapes subsequent stress exposure.

For independent negative life events, no within‐person effect on life satisfaction was observed over the 2‐year intervals in either activity group. However, group differences emerged for the reversed pathway: among physically active individuals, higher‐than‐usual life satisfaction was associated with a lower likelihood of subsequent independent events, whereas lower‐than‐usual life satisfaction increased this likelihood. Furthermore, among active individuals, reductions in independent negative life events were associated with a lower likelihood of subsequent dependent stressors, indicating cascading protective effects across stressor domains.

At the between‐person level, individuals experiencing more dependent negative life events reported lower overall life satisfaction in both activity groups. Independent negative life events were negatively associated with life satisfaction only among individuals with low physical activity, whereas active individuals showed no such association. This could be interpreted such that physical activity buffers sustained differences in well‐being linked to repeated exposure to largely uncontrollable stressors.

## 6 General Discussion

The present study aimed to clarify the buffering effect of physical activity on the association between negative life events and life satisfaction, by explicitly distinguishing between dependent and independent stressors, within‐person and between‐person processes, and short‐ (1‐year interval) versus medium‐term (2‐year interval) dynamics. Drawing on stress generation theory (Hammen 1991; Liu et al. 2024; Rnic et al. 2023) and set‐point theory (Headey and Wearing 1989; Lykken and Tellegen 1996), the study extends prior work using an analytical framework comparable to recent research on volunteering (Groß and Haffa 2026). Thus, the present study enables a direct comparison between resilience resources (Nichol et al. 2024; Rahmati et al. 2024), namely physical activity and volunteering.

### 6.1 Within‐Person Effects of Dependent Negative Life Events on Life Satisfaction

Across both datasets, dependent negative life events—those which are assumed to be closely linked to individuals' behaviours and decisions (Maciejewski et al. 2021)—were often associated with subsequent declines in life satisfaction at the within‐person level. However, the strength of this association varied across physical activity groups and time intervals. The association was consistently observed among individuals with low levels of physical activity across both time intervals. In contrast, among individuals with high levels of physical activity, the association was attenuated over 2‐year intervals, such that dependent negative life events no longer predicted subsequent declines in life satisfaction. These findings suggest that physical activity may buffer the medium‐term impact of behaviour‐linked stressors on well‐being, although this buffering effect does not appear to emerge immediately. Over 1‐year intervals, no comparable attenuation was observed, indicating that short‐term within‐person stress reactivity remains relatively robust even among physically active individuals.

A similar temporal pattern was reported by Groß and Haffa (2026) for volunteering, suggesting that behavioural resilience resources may require longer temporal intervals before protective effects become observable at the within‐person level. One possible explanation is that regulatory and coping‐related adaptations linked to physical activity accumulate gradually over time and therefore become more visible across longer observation windows. Importantly, the findings indicate a time‐dependent attenuation rather than a uniform buffering process. Physical activity did not eliminate within‐person associations between dependent stressors and life satisfaction across 1‐year intervals but appeared to weaken these associations specifically over 2‐year intervals.

### 6.2 Within‐Person Effects of Life Satisfaction on Dependent Negative Life Events

Across both datasets and physical activity groups, within‐person deviations in life satisfaction were consistently negatively associated with subsequent dependent negative life events. Specifically, lower‐than‐usual life satisfaction predicted an increased likelihood of subsequent dependent stressors, whereas higher‐than‐usual life satisfaction predicted a reduced likelihood of later stress exposure. This pattern is consistent with stress generation theory (Hammen 1991; Liu et al. 2024; Rnic et al. 2023), which proposes that lower well‐being may contribute to behaviours, coping styles, and interpersonal processes that increase subsequent stress exposure. Contrary to expectations, physical activity did not consistently attenuate this pathway across time intervals. Thus, although physical activity buffered the consequences of dependent stressors for life satisfaction over 2‐year intervals, it did not similarly weaken the reverse prospective association from life satisfaction to subsequent dependent stress exposure. This asymmetry suggests that physical activity may primarily support emotional recovery and stress regulation following stressful experiences rather than fundamentally altering the interpersonal and behavioural processes underlying stress generation. This interpretation is contrasted by findings from Groß and Haffa (2026), who reported that volunteering attenuated this pathway over medium‐term intervals. Compared to physical activity, volunteering may more directly influence interpersonal integration, social role functioning, and social support processes, which are particularly relevant for dependent stress generation mechanisms. Accordingly, different behavioural resilience resources may operate through partly distinct mechanisms.

### 6.3 Within‐Person Effects of Independent Negative Life Events on Life Satisfaction

For independent negative life events, the findings revealed an unexpected pattern across physical activity groups and time intervals. In the HILDA data, over 1‐year intervals, independent negative life events predicted subsequent declines in life satisfaction among physically active individuals, whereas no such association emerged among those with low physical activity. Thus, contrary to expectations, physical activity did not buffer within‐person association between independent stressors and life satisfaction in the short term. One possible explanation is that independent negative life events—such as bereavement, serious illness, or victimization—may temporarily disrupt the benefits typically associated with physical activity. Although physically active individuals may maintain activity through habitual routines, severe uncontrollable stressors may reduce the affective and motivational benefits usually linked to physical activity engagement. Consistent with self‐determination theory and prior findings (Meyer et al. 2021; Ryan and Deci 2000), physical activity may not confer stress‐buffering effects when stress undermines intrinsic motivation.

In contrast, in the SOEP data across 2‐year intervals, independent negative life events no longer predicted subsequent declines in life satisfaction in either physical activity group. This pattern is consistent with set‐point theory, which assumes gradual recovery towards baseline well‐being following uncontrollable stressors (Headey and Wearing 1989; Lykken and Tellegen 1996). Importantly, adaption processes appeared to emerge across both activity groups over medium‐term intervals, indicating that physical activity did not confer additional advantages once adaptation processes had unfolded over time.

These findings differ from those reported by Groß and Haffa (2026), who observed short‐term buffering effects of volunteering for independent negative life events. One possible interpretation is, as noted above, that physical activity and volunteering represent distinct resilience resources that operate through partly different mechanisms. Whereas volunteering may provide more immediate social and emotional resources during stressful periods, physical activity may be more sensitive to contextual and motivational disruptions following severe uncontrollable events.

Overall, the findings suggest that within‐person associations between independent negative life events and life satisfaction are shaped by both temporal structure and behavioural context. Short‐term coupling differed across physical activity groups, whereas over longer intervals both groups converged towards attenuated associations consistent with adaptation processes, which were expected due to set‐point theory (Headey and Wearing 1989; Lykken and Tellegen 1996).

### 6.4 Within‐Person Effects of Life Satisfaction on Independent Negative Life Events

Among individuals with low levels of physical activity, life satisfaction did not predict subsequent independent events across either time interval, consistent with the largely uncontrollable and exogenous nature of these stressors. In contrast, among physically active individuals, lower‐than‐usual life satisfaction predicted a higher likelihood of subsequent independent negative life events, whereas higher‐than‐usual life satisfaction predicted a lower likelihood of subsequent independent negative life events. Importantly, these findings should not be interpreted as evidence that life satisfaction directly influences rather uncontrollable events such as bereavement or serious illness. Rather, they likely reflect broader contextual and behavioural processes associated with physical activity and well‐being. Physically active individuals are often embedded in health‐promoting social and behavioural environments characterised by healthier lifestyles, stronger social integration, and lower morbidity risks (Carr et al. 2018; Pauly et al. 2020; Prochnow and Patterson 2022). Because several assessed independent negative life events relate to health, safety, and close relationships, fluctuations in life satisfaction within these contexts may indirectly correspond to variations in exposure risk over time.

Notably, Groß and Haffa (2026) reported a different pattern for volunteering, with associations emerging across both volunteering groups at shorter intervals but disappearing over longer intervals. Together, these findings suggest that associations between life satisfaction and subsequent independent stressors vary not only across time but also across behavioural contexts. Physical activity appears to be associated with between‐person heterogeneity (low vs. high physical active) in stress‐related coupling processes, whereas volunteering primarily influences the temporal structure (one vs. two‐year) of these associations.

### 6.5 Within‐Person Effects Between Independent Negative Life Events and Dependent Negative Life Events

In the SOEP data with 2‐year intervals, within‐person reductions in independent negative life events were associated with a lower likelihood of subsequent dependent negative life events among physically active individuals. This pattern suggests a potential cross‐domain association between independent and dependent stressors at the within‐person level. Specifically, periods characterised by fewer independent stressors may be linked to more stable behavioural and contextual conditions among physically active individuals, which in turn coincide with a reduced likelihood of subsequent dependent stressors. However, given the observational nature of this finding, this interpretation remains tentative. No comparable cross‐domain association was observed in the volunteering study (Groß and Haffa 2026), indicating that such patterns may depend on physical activity. This suggests that physical activity and volunteering may differ in how they relate to the temporal coupling of distinct stressor domains, although further research is needed to clarify the robustness and mechanisms underlying these differences.

### 6.6 Autoregressive Effects of Independent Negative Life Events

Beyond the adaptation processes predicted by set‐point theory (Headey and Wearing 1989; Lykken and Tellegen 1996), differences emerged in the temporal stability of independent negative life events. In the SOEP data with 2‐year intervals, the autoregressive stability of independent negative life events was reduced among physically active individuals, whereas no such pattern was observed among individuals with low physical activity. This finding suggests that physical activity may be associated with broader contextual and behavioural conditions that influence the temporal clustering of independent stressors. Physically active individuals are more likely to be embedded in health‐oriented social environments characterised by shared routines and mutual health‐related behaviours (Carr et al. 2018; Pauly et al. 2020; Prochnow and Patterson 2022), which may contribute to more stable life conditions and reduced persistence of stress exposure over time. Importantly, this pattern does not imply that physical activity influences the occurrence of inherently uncontrollable events. Rather, it may reflect indirect and cumulative processes through which physical activity is associated with differences in living conditions, social embedding, and health trajectories. A similar pattern was observed in the volunteering study by Groß and Haffa (2026), where reduced autoregressive stability of independent life events was also found over medium‐term intervals. Taken together, these findings suggest that behavioural resources may influence not only stress reactivity but also the temporal structure of stress exposure, although through indirect and context‐dependent pathways.

### 6.7 Between‐Person Associations

At the between‐person level, dependent and independent negative life events were positively correlated in both physical activity groups over the 1‐year intervals, whereas this association was not evident over the 2‐year intervals. This pattern suggests that co‐occurrence of different stressor types is more pronounced in shorter temporal windows, while between‐person differences become more differentiated when observed over longer time spans.

For independent negative life events, a buffering effect of physical activity on life satisfaction was observed only in the 2‐year interval (SOEP). In contrast, no such moderation emerged in the 1‐year interval (HILDA). This pattern is consistent with set‐point theory (Headey and Wearing 1989; Lykken and Tellegen 1996), which posits that independent negative life events induce temporary deviations in well‐being followed by gradual adaptation towards baseline levels over time. Accordingly, between‐person differences are conceptualised as time‐aggregated average manifestations, which only become observable once sufficient temporal accumulation has occurred.

Importantly, this does not imply that adaptation differs fundamentally between physical activity groups. Rather, it suggests that between‐person associations reflect different degrees of temporal aggregation. In this sense, the emergence of a buffering effect in the 2‐year interval may indicate that these aggregated patterns become detectable earlier or more clearly among physically active individuals.

In contrast, dependent negative life events were consistently associated with lower life satisfaction across both physical activity groups and both time intervals, with only minor attenuation over time and no evidence of moderation by physical activity. This pattern aligns with stress generation theory, which conceptualises dependent stressors as arising from stable person–environment transactions in which affective functioning, coping behaviour, and interpersonal processes jointly contribute to stress exposure over time (Hammen 1991; Liu et al. 2024; Rnic et al. 2023). As such, these associations reflect relatively stable, self‐reinforcing coupling processes that are less sensitive to behavioural buffering resources such as physical activity. This pattern is broadly consistent with prior research on behavioural resilience resources such as volunteering (Groß and Haffa 2026), suggesting that such resources primarily operate at the level of within‐person dynamics, whereas between‐person associations reflect longer‐term structural patterns shaped by both stressor type and temporal aggregation.

### 6.8 Integrating These Findings

Taken together, the findings underscore that physical activity operates as a conditional resilience factor whose effects depend on the type of stressor (dependent vs. independent negative life events), the direction of the stress process (stress → well‐being vs. well‐being → stress), the analysis level (between‐person or within‐person) and the temporal scale of observation. Rather than exerting a uniform buffering effect, physical activity shapes stress dynamics in a differentiated manner across stressor domains, time horizons, and levels of analysis. Regarding stress‐to‐well‐being processes, dependent negative life events predicted subsequent declines in life satisfaction, with evidence of moderation by physical activity in the medium‐term interval. In contrast, short‐term buffering effects were less consistent including for independent negative life events, indicating that protective effects may require time to accumulate. For well‐being‐to‐stress processes, higher life satisfaction was generally associated with a subsequent reduction in stress exposure. This pattern was evident for dependent stressors irrespective of physical activity, and, among physically active individuals, also for independent stressors across both time intervals. Taken together, these findings suggest that physical activity does not uniformly buffer stress processes, but instead operates through conditionally influenced mechanisms that vary by stressor type, directional pathway, and temporal scale. This multidimensional structure helps reconcile mixed evidence in prior research (e.g., Gerber and Pühse 2009; Klaperski 2018) by demonstrating that buffering effects are not generalisable across all contexts but emerge only under specific combinations of stressor‐well‐being characteristics and time horizons.

### 6.9 Practical Implications

The present findings have several implications for interventions and public health strategies aimed at promoting resilience through physical activity. First, the results suggest that physical activity should not be conceptualised as a universally effective buffer against stress, but rather as a context‐dependent resource whose benefits vary by stressor type, directional process, and time horizon. This implies that intervention strategies may be more effective when they are tailored to specific stress contexts and time intervals, rather than assuming uniform protective effects across individuals and situations.

Second, the findings highlight the importance of maintaining physical activity during periods of elevated stress, as short‐term disruptions in motivational quality or perceived benefits may attenuate its protective effects. Interventions that support sustained engagement—particularly through the promotion of intrinsic motivation (cf. Meyer et al. 2021)—may therefore be especially relevant, consistent with self‐determination theory (Ryan and Deci 2000).

Third, the results suggest that physical activity is embedded within broader social and environmental contexts that may jointly influence both stress exposure and coping capacity. Accordingly, interventions that integrate social components—particularly dyadic or group‐based physical activity interventions involving partners, peers, or community networks—may enhance physical activity level (cf. Carr et al. 2018).

Finally, the observed temporal dynamics indicate that the effects of physical activity are unlikely to manifest immediately and may unfold over longer time horizons. However, in the context of independent negative life events, the results are consistent with general adaptation processes towards baseline levels of life satisfaction that appear to operate largely independently of physical activity (cf. set‐point theory; Headey and Wearing 1989; Lykken and Tellegen 1996).

### 6.10 Limitations and Future Studies

Several limitations should be acknowledged. First, the study relies on retrospective self‐reports, which are vulnerable to recall bias, reporting inaccuracies, and social desirability effects (Adams et al. 2005). In addition, the precise timing of negative life events within assessment intervals could not be determined. As the psychological impact of stressors may depend on their recency, future studies should examine whether the buffering role of physical activity varies as a function of temporal proximity.

Second, limitations arise from the measurement of negative life events in the SOEP. Events were recorded only when explicitly endorsed, while missing or non‐applicable responses were recoded as non‐occurrence. Consequently, sum scores likely represent conservative estimates, potentially underestimating the frequency and stability of negative life events.

Third, although RI‐CLPMs are well suited to disentangle within‐ and between‐person processes, they assume linear dynamics and are sensitive to the length of measurement intervals (Kuiper and Ryan 2018). While using two interval lengths is a strength, future research should more systematically vary intervals to identify the time windows in which physical activity exerts its strongest buffering effects.

Another limitation concerns the operationalisation of physical activity as a time‐invariant categorical moderator based on median engagement across waves. This static classification may obscure meaningful within‐person fluctuations. Future studies should use continuous, time‐varying measures of physical activity (e.g., Ozkok et al. 2022; Speyer et al. 2023) and consider motivational quality, as stress‐buffering effects may depend on intrinsic motivation (Meyer et al. 2021).

A further limitation involves the classification of negative life events into dependent and independent categories. Although theoretically grounded, this dichotomy oversimplifies perceived controllability, which likely varies across individuals and contexts. Incorporating subjective appraisals of controllability, severity, and chronicity would provide a more refined understanding of stress‐buffering mechanisms.

The use of a single‐item measure of life satisfaction also limits the assessment of multidimensional well‐being, despite acceptable psychometric evidence (Cheung and Lucas 2014). Finally, generalisability may be limited, as the samples stem from Western, high‐income countries; future research should examine whether these findings extend to other cultural and institutional contexts.

## 7 Conclusion

This synthesis of two panel studies shows that the associations between negative life events and life satisfaction are highly conditional and depend on stressor type, temporal scale, and level of analysis. Dependent negative life events predicted declines in life satisfaction, with evidence of conditional buffering effects of physical activity over longer time intervals, whereas short‐term effects were less pronounced. In contrast, independent negative life events showed no consistent medium‐term impact on life satisfaction, consistent with set‐point theory (Headey and Wearing 1989; Lykken and Tellegen 1996) and the general adaptation processes towards baseline levels of well‐being. Contrary to expectations, short‐term declines in life satisfaction following independent negative life events were observed only among physically active individuals in the 1‐year interval, whereas no such association emerged among individuals with low physical activity. This pattern suggests that short‐term buffering effects of physical activity may be disrupted under conditions of severe uncontrollable stress, while low activity groups may already reflect more stable within‐person dynamics consistent with early adaptation processes.

In the reverse direction, within‐person reduction in life satisfaction were associated with subsequent stress exposure, particularly for dependent negative life events. However, within the RI‐CLPM framework, these associations should be interpreted as bidirectionally consistent coupling processes rather than unidirectional effects, and their direction of influence cannot be definitively established.

Importantly, the moderating role of physical activity differed across levels of analysis: at the within‐person level, moderation effects were primarily observed for dependent negative life events, whereas at the between‐person level, associations were more pronounced for independent negative life events.

Taken together with prior evidence on volunteering (Groß and Haffa 2026), the findings suggest that resilience resources do not exert uniform buffering effects, but operate within specific temporal, contextual, and structural boundaries. Overall, physical activity emerges as a conditional resilience factor that shapes stress dynamics in a level‐specific and stressor‐dependent manner.

## Ethics Statement

The responsible institution obtained informed consent from participants. Ethical approval was not required for the current research, as we analysed existing and fully anonymised panel data.

## Conflicts of Interest

All authors declare no competing interests.

## Supporting information


Supporting Information S1

## Data Availability

The analysis code is provided on the Open Science Framework (OSF) and can be retrieved from https://osf.io/qyb34. On OSF, we share the data analytic methods and codes necessary to reproduce our results directly from the original data sets without the need for any additional steps to prepare the data. The data sets used in this study are made available through the respective data‐holding institutions. In accordance to regulations of these data‐holding institution, we are not allowed to make the data publicly available. This paper uses unit record data from the Household, Income and Labour Dynamics in Australia Survey [HILDA] conducted by the Australian Government Department of Social Services (DSS). The findings and views reported in this paper, however, are those of the authors and should not be attributed to the Australian Government, DSS, or any of DSS’ contractors or partners (https://dataverse.ada.edu.au/dataset.xhtml?persistentId=doi:10.26193/KXNEBO). Additionally, this paper uses data from the Socio‐Economic Panel (SOEP) made available by the German Institute for Economic Research (DIW).
