# Supplementary material for: Buffering Effects of Physical Activity on Negative Life Events–Life Satisfaction Dynamics
Source: Stress Health. 2026 Jul 1;42(4):e70198. doi: 10.1002/smi.70198 (PMC13324232; doi:10.1002/smi.70198)
Supplement: Supplementary file 1 — Supporting Information S1 [file SMI-42-e70198-s001.docx]

**Random Intercept Cross-Lagged Panel Model (RI-CLPM)**

The RI-CLPM extends the traditional cross-lagged panel model by including random intercepts that capture stable between-person differences, thereby allowing a clear separation of within-person temporal dynamics from between-person associations (Hamaker et al., 2015; Mund & Nestler, 2019). In the RI-CLPM, correlations between random intercept factors primarily reflect between-person associations derived from information across multiple waves (e.g., individuals who experience more independent negative life events are less satisfied when being less active individuals). Within-person autoregressive effects reflect the temporal stability of a variable within an individual. For example, a positive autoregressive effect indicates that if a person scores above their average level of life satisfaction at one time point, they are likely to score above their average at the next time point as well. Within-person cross-lagged effects refer to the temporal influence of one variable at *t* on another variable at *t*+1.

**Model Constraints and Model Comparison**

Before conducting multiple-group comparisons, we tested the tenability of time-invariant autoregressive and cross-lagged parameters for each panel (Mulder & Hamaker, 2021). Specifically, we compared the fit of a multiple-group model assuming time-invariant autoregressive and cross-lagged parameters (with no constraints across physical activity groups) to a corresponding model in which these parameters were freely estimated over time.

Model comparisons were based on the Akaike Information Criterion (AIC) and the Bayesian Information Criterion (BIC), as observations were equally spaced within each panel (one-year intervals in HILDA and two-year intervals in SOEP; Preacher & Yaremych, 2023). The AIC prioritizes predictive accuracy, whereas the BIC emphasizes model parsimony and consistency. In both panels, the AIC favored models without time-invariance constraints, whereas the BIC favored models with constrained autoregressive and cross-lagged parameters. Given the BIC’s stronger penalty for model complexity, we assumed time-invariant autoregressive and cross-lagged parameters in all subsequent analyses.

To examine moderation by physical activity, we then compared this baseline model to a more constrained multiple-group model in which all parameters – including autoregressive and cross-lagged effects – were constrained to be equal across the low and high physical activity groups. Differences in model fit were evaluated using the BIC, with lower values indicating better fit.

**Missing Data**

Given the longitudinal nature of the panel studies, missing data were expected at both the item and wave levels. Accordingly, all individuals who participated in at least one wave were included in the analyses. Missing data were handled using Full Information Maximum Likelihood (FIML) estimation, which yields unbiased parameter estimates under the assumption that data are missing at random (MAR). Under MAR, the probability of missingness may depend on other observed variables in the dataset but not on the unobserved values themselves (Enders, 2010). To further strengthen the plausibility of the MAR assumption and enhance the robustness of the FIML estimation, participants’ age at T1 was included as an auxiliary variable, in line with established recommendations (Enders, 2008; Graham, 2003).

Since FIML can only be applied to continuous variables, gender could not be used as an auxiliary variable. However, within-person coefficient estimates remain unaffected by time-invariant covariates like gender (Usami et al., 2019).

**Study 1: The Household, Income and Labour Dynamics in Australia (HILDA)**

***Measures***

***Independent negative life events.*** Individuals were asked whether they had experienced the following independent negative life events in the past year: death of a close friend, death of close relative/family member, death of spouse or child, serious injury/illness to family member, serious personal injury/illness, close family member detained in jail, victim of a property crime, victim of physical violence. In the original data set, the presence of each life event was coded as 2, and absence as 1. For the present analyses, responses were re-coded such that presence was coded as 1, and absence as 0. A sum score was calculated.

***Dependent negative life events.*** Individuals were asked whether they had experienced the following dependent negative life events in the past year: major worsening in finances, fired or made redundant by an employer, detained in jail, separated from spouse. In the original data set, the presence of each life event was coded as 2, and absence as 1. For the present analyses, responses were re-coded such that presence was coded as 1, and absence as 0. A sum score was calculated.

***Life satisfaction.*** Life satisfaction was also measured with one item: “All things considered, how satisfied are you with your life?” with the following response options: (0) *totally dissatisfied* to (10) *totally satisfied*.

***Moderate-to-vigorous physical activity.*** Physical activity was measured with one item: “In general, how often do you participate in moderate or intensive physical activity for at least 30 minutes?” with the following response options: (1) *not at all*, (2) *less than once a week*, (3) *1 to 2 times a week*, (4) *3 times a week*, (5) *more than 3 times a week*, (6) *every day*. From 2020, the question contained the following addition: “Moderate level physical activity will cause a slight increase in breathing and heart rate, such as brisk walking.” Before 2020, no explicit instruction was given with respect to what participants should consider as moderate or intensive physical activity. To categorize individuals into low or high levels of physical activity, we calculated each person's median physical activity across all waves in which they participated. Those who engaged in moderate-to-vigorous physical activity generally less than once a week were assigned to the low physical activity group (0), while those who participated at least once a week were assigned to the high physical activity group (1).

**Study 2: German Socio-Economic Panel (SOEP)**

***Measures***

***Independent negative life events.*** Participants were asked whether they had experienced any of the following negative life events in the past year: spouse/partner died, father died, mother died, or child died. In the SOEP, these events are coded as 1 = yes, with all other responses representing missing or non-applicable values (e.g., −1 = no answer, −2 = does not apply, −5 = not included in this version). Because no explicit “no” category exists, events were coded as 1 when reported and 0 otherwise, indicating that the event was not observed. A sum score was then calculated to reflect the number of events experienced.

***Dependent negative life events.*** Participants were asked whether they had experienced the following dependent negative life events in the past year: separation from a spouse/partner and divorce. In the SOEP, these events are coded as 1 = yes, with all other responses representing missing or non-applicable values. For the current analysis, events were coded as 1 when reported and 0 otherwise, indicating that the event was not observed. Additionally, official unemployment status was included in the sum score: in the original dataset, 2 = unemployed and 1 = not unemployed; for the present analysis, these values were recoded so that 1 = event occurred (unemployed) and 0 = event did not occur (not unemployed). A total sum score was then calculated to reflect the number of dependent negative life events experienced.

***Life satisfaction.*** Life satisfaction was also measured with one item: “How satisfied are you with your life, all things considered?” with the following response options: (0) *completely dissatisfied* to (10) *completely satisfied*.

***Sport participation in leisure time.*** Physical activity was measured with one item: “Now some questions about your leisure time. Please indicate how often you take part in active sports?” with the following response options: (1) *daily*, (2) *at least once per week*, (3) *at least once per month*, (4) *seldom*, (5) *never*. The responses to this item were inverted so that a higher value corresponded to higher physical activity. Hence, the assessment was based on a broad and subjective understanding of sport and sport activity in leisure time as a measure of physical activity. It should be noted that physical activity is more than only sport participation in leisure time since it additionally includes occupational activities, conditioning exercises, household tasks (for example, yardwork, cleaning, and home repair), and other activities (Caspersen et al., 1985). To categorize individuals into low or high levels of physical activity, we calculated each person's median physical activity across all waves in which they participated. Those who engaged in sport participation during leisure time less than once a week were assigned to the low physical activity group (0), while those who participated at least once a week were assigned to the high physical activity group (1).

| **Table 1**  *Descriptive Statistics for Mean Age, Gender, and the Distributions of Independent Negative Life Events, Dependent Negative Life Events, and Life Satisfaction across all Time Points and by Group* *– HILDA Data* | | | | | | | | | | |
| --- | --- | --- | --- | --- | --- | --- | --- | --- | --- | --- |
| Variable | Wave | n_Missing | Complete Rate | *M* | *SD* | 0th Percentile | 25th Percentile | 50th Percentile | 75th Percentile | 100th Percentile |
| **Low PA** |  |  |  |  |  |  |  |  |  |  |
| IN_NLE | 1 | 4592 | 0.40 | 0.61 | 0.84 | 0.00 | 0.00 | 0.00 | 1.00 | 5.00 |
| IN_NLE | 2 | 5069 | 0.34 | 0.65 | 0.88 | 0.00 | 0.00 | 0.00 | 1.00 | 6.00 |
| IN_NLE | 3 | 4889 | 0.36 | 0.65 | 0.89 | 0.00 | 0.00 | 0.00 | 1.00 | 8.00 |
| IN_NLE | 4 | 4931 | 0.36 | 0.61 | 0.87 | 0.00 | 0.00 | 0.00 | 1.00 | 6.00 |
| IN_NLE | 5 | 4958 | 0.35 | 0.64 | 0.90 | 0.00 | 0.00 | 0.00 | 1.00 | 8.00 |
| IN_NLE | 6 | 5063 | 0.34 | 0.56 | 0.82 | 0.00 | 0.00 | 0.00 | 1.00 | 5.00 |
| IN_NLE | 7 | 5174 | 0.33 | 0.57 | 0.82 | 0.00 | 0.00 | 0.00 | 1.00 | 6.00 |
| IN_NLE | 8 | 5103 | 0.33 | 0.59 | 0.83 | 0.00 | 0.00 | 0.00 | 1.00 | 5.00 |
| IN_NLE | 9 | 4969 | 0.35 | 0.59 | 0.83 | 0.00 | 0.00 | 0.00 | 1.00 | 5.00 |
| IN_NLE | 10 | 4111 | 0.46 | 0.59 | 0.85 | 0.00 | 0.00 | 0.00 | 1.00 | 6.00 |
| IN_NLE | 11 | 4156 | 0.46 | 0.64 | 0.90 | 0.00 | 0.00 | 0.00 | 1.00 | 6.00 |
| IN_NLE | 12 | 4194 | 0.45 | 0.59 | 0.85 | 0.00 | 0.00 | 0.00 | 1.00 | 8.00 |
| IN_NLE | 13 | 4219 | 0.45 | 0.60 | 0.86 | 0.00 | 0.00 | 0.00 | 1.00 | 5.00 |
| IN_NLE | 14 | 4245 | 0.45 | 0.60 | 0.85 | 0.00 | 0.00 | 0.00 | 1.00 | 7.00 |
| IN_NLE | 15 | 4098 | 0.47 | 0.64 | 0.91 | 0.00 | 0.00 | 0.00 | 1.00 | 8.00 |
| IN_NLE | 16 | 4189 | 0.45 | 0.55 | 0.83 | 0.00 | 0.00 | 0.00 | 1.00 | 6.00 |
| IN_NLE | 17 | 4224 | 0.45 | 0.58 | 0.85 | 0.00 | 0.00 | 0.00 | 1.00 | 7.00 |
| IN_NLE | 18 | 4203 | 0.45 | 0.59 | 0.90 | 0.00 | 0.00 | 0.00 | 1.00 | 8.00 |
| IN_NLE | 19 | 4294 | 0.44 | 0.61 | 0.91 | 0.00 | 0.00 | 0.00 | 1.00 | 6.00 |
| IN_NLE | 20 | 4420 | 0.42 | 0.55 | 0.83 | 0.00 | 0.00 | 0.00 | 1.00 | 6.00 |
| IN_NLE | 21 | 4532 | 0.41 | 0.63 | 0.90 | 0.00 | 0.00 | 0.00 | 1.00 | 6.00 |
| DE_NLE | 1 | 4556 | 0.41 | 0.12 | 0.38 | 0.00 | 0.00 | 0.00 | 0.00 | 3.00 |
| DE_NLE | 2 | 5055 | 0.34 | 0.11 | 0.35 | 0.00 | 0.00 | 0.00 | 0.00 | 3.00 |
| DE_NLE | 3 | 4864 | 0.37 | 0.11 | 0.36 | 0.00 | 0.00 | 0.00 | 0.00 | 4.00 |
| DE_NLE | 4 | 4901 | 0.36 | 0.11 | 0.35 | 0.00 | 0.00 | 0.00 | 0.00 | 3.00 |
| DE_NLE | 5 | 4951 | 0.35 | 0.11 | 0.36 | 0.00 | 0.00 | 0.00 | 0.00 | 4.00 |
| DE_NLE | 6 | 5044 | 0.34 | 0.11 | 0.35 | 0.00 | 0.00 | 0.00 | 0.00 | 3.00 |
| DE_NLE | 7 | 5166 | 0.33 | 0.11 | 0.36 | 0.00 | 0.00 | 0.00 | 0.00 | 3.00 |
| DE_NLE | 8 | 5075 | 0.34 | 0.13 | 0.39 | 0.00 | 0.00 | 0.00 | 0.00 | 3.00 |
| DE_NLE | 9 | 4953 | 0.35 | 0.12 | 0.36 | 0.00 | 0.00 | 0.00 | 0.00 | 3.00 |
| DE_NLE | 10 | 4076 | 0.47 | 0.12 | 0.36 | 0.00 | 0.00 | 0.00 | 0.00 | 3.00 |
| DE_NLE | 11 | 4113 | 0.46 | 0.11 | 0.36 | 0.00 | 0.00 | 0.00 | 0.00 | 3.00 |
| DE_NLE | 12 | 4166 | 0.46 | 0.12 | 0.38 | 0.00 | 0.00 | 0.00 | 0.00 | 3.00 |
| DE_NLE | 13 | 4162 | 0.46 | 0.11 | 0.35 | 0.00 | 0.00 | 0.00 | 0.00 | 3.00 |
| DE_NLE | 14 | 4210 | 0.45 | 0.12 | 0.37 | 0.00 | 0.00 | 0.00 | 0.00 | 3.00 |
| DE_NLE | 15 | 4062 | 0.47 | 0.11 | 0.35 | 0.00 | 0.00 | 0.00 | 0.00 | 3.00 |
| DE_NLE | 16 | 4148 | 0.46 | 0.09 | 0.31 | 0.00 | 0.00 | 0.00 | 0.00 | 2.00 |
| DE_NLE | 17 | 4178 | 0.46 | 0.09 | 0.31 | 0.00 | 0.00 | 0.00 | 0.00 | 3.00 |
| DE_NLE | 18 | 4184 | 0.45 | 0.11 | 0.37 | 0.00 | 0.00 | 0.00 | 0.00 | 4.00 |
| DE_NLE | 19 | 4285 | 0.44 | 0.12 | 0.39 | 0.00 | 0.00 | 0.00 | 0.00 | 4.00 |
| DE_NLE | 20 | 4415 | 0.42 | 0.10 | 0.32 | 0.00 | 0.00 | 0.00 | 0.00 | 3.00 |
| DE_NLE | 21 | 4520 | 0.41 | 0.09 | 0.33 | 0.00 | 0.00 | 0.00 | 0.00 | 3.00 |
| LIFE | 1 | 4438 | 0.42 | 7.68 | 1.81 | 0.00 | 7.00 | 8.00 | 9.00 | 10.00 |
| LIFE | 2 | 4956 | 0.35 | 7.80 | 1.73 | 0.00 | 7.00 | 8.00 | 9.00 | 10.00 |
| LIFE | 3 | 4775 | 0.38 | 7.76 | 1.72 | 0.00 | 7.00 | 8.00 | 9.00 | 10.00 |
| LIFE | 4 | 4818 | 0.37 | 7.66 | 1.70 | 0.00 | 7.00 | 8.00 | 9.00 | 10.00 |
| LIFE | 5 | 4848 | 0.37 | 7.66 | 1.71 | 0.00 | 7.00 | 8.00 | 9.00 | 10.00 |
| LIFE | 6 | 4950 | 0.35 | 7.66 | 1.68 | 0.00 | 7.00 | 8.00 | 9.00 | 10.00 |
| LIFE | 7 | 5073 | 0.34 | 7.66 | 1.63 | 0.00 | 7.00 | 8.00 | 9.00 | 10.00 |
| LIFE | 8 | 5011 | 0.35 | 7.63 | 1.67 | 0.00 | 7.00 | 8.00 | 9.00 | 10.00 |
| LIFE | 9 | 4884 | 0.36 | 7.60 | 1.67 | 0.00 | 7.00 | 8.00 | 9.00 | 10.00 |
| LIFE | 10 | 4002 | 0.48 | 7.65 | 1.70 | 0.00 | 7.00 | 8.00 | 9.00 | 10.00 |
| LIFE | 11 | 4052 | 0.47 | 7.67 | 1.65 | 0.00 | 7.00 | 8.00 | 9.00 | 10.00 |
| LIFE | 12 | 4091 | 0.47 | 7.63 | 1.67 | 0.00 | 7.00 | 8.00 | 9.00 | 10.00 |
| LIFE | 13 | 4097 | 0.47 | 7.64 | 1.68 | 0.00 | 7.00 | 8.00 | 9.00 | 10.00 |
| LIFE | 14 | 4130 | 0.46 | 7.60 | 1.75 | 0.00 | 7.00 | 8.00 | 9.00 | 10.00 |
| LIFE | 15 | 3991 | 0.48 | 7.63 | 1.66 | 0.00 | 7.00 | 8.00 | 9.00 | 10.00 |
| LIFE | 16 | 4081 | 0.47 | 7.58 | 1.77 | 0.00 | 7.00 | 8.00 | 9.00 | 10.00 |
| LIFE | 17 | 4126 | 0.46 | 7.66 | 1.64 | 0.00 | 7.00 | 8.00 | 9.00 | 10.00 |
| LIFE | 18 | 4110 | 0.46 | 7.68 | 1.65 | 0.00 | 7.00 | 8.00 | 9.00 | 10.00 |
| LIFE | 19 | 4223 | 0.45 | 7.74 | 1.57 | 0.00 | 7.00 | 8.00 | 9.00 | 10.00 |
| LIFE | 20 | 4343 | 0.43 | 7.73 | 1.55 | 0.00 | 7.00 | 8.00 | 9.00 | 10.00 |
| LIFE | 21 | 4453 | 0.42 | 7.76 | 1.57 | 0.00 | 7.00 | 8.00 | 9.00 | 10.00 |
| Age | 1 | 4437 | 0.42 | 48.58 | 18.56 | 15.00 | 34.00 | 46.00 | 63.00 | 94.00 |
| Age | 2 | 4955 | 0.35 | 50.05 | 18.20 | 16.00 | 36.00 | 48.00 | 64.00 | 94.00 |
| Age | 3 | 4774 | 0.38 | 49.04 | 18.61 | 15.00 | 35.00 | 47.00 | 63.00 | 97.00 |
| Age | 4 | 4817 | 0.37 | 48.95 | 18.80 | 15.00 | 35.00 | 47.00 | 63.00 | 94.00 |
| Age | 5 | 4847 | 0.37 | 49.33 | 18.76 | 15.00 | 35.25 | 48.00 | 63.00 | 96.00 |
| Age | 6 | 4950 | 0.35 | 49.69 | 18.91 | 15.00 | 36.00 | 48.00 | 63.50 | 97.00 |
| Age | 7 | 5073 | 0.34 | 49.84 | 18.72 | 15.00 | 36.00 | 49.00 | 63.00 | 96.00 |
| Age | 8 | 5008 | 0.35 | 49.66 | 18.79 | 15.00 | 36.00 | 49.00 | 63.00 | 98.00 |
| Age | 9 | 4883 | 0.36 | 49.25 | 18.68 | 15.00 | 36.00 | 48.00 | 62.00 | 98.00 |
| Age | 10 | 4002 | 0.48 | 49.47 | 18.77 | 15.00 | 35.00 | 49.00 | 63.00 | 99.00 |
| Age | 11 | 4049 | 0.47 | 49.58 | 18.68 | 15.00 | 36.00 | 49.00 | 63.00 | 100.00 |
| Age | 12 | 4091 | 0.47 | 49.77 | 18.72 | 15.00 | 35.00 | 49.00 | 63.00 | 101.00 |
| Age | 13 | 4095 | 0.47 | 50.09 | 18.69 | 15.00 | 36.00 | 50.00 | 64.00 | 97.00 |
| Age | 14 | 4130 | 0.46 | 50.18 | 18.77 | 15.00 | 36.00 | 50.00 | 64.00 | 97.00 |
| Age | 15 | 3991 | 0.48 | 50.07 | 18.72 | 15.00 | 35.00 | 50.00 | 63.00 | 98.00 |
| Age | 16 | 4080 | 0.47 | 50.10 | 18.71 | 15.00 | 35.00 | 50.00 | 63.00 | 99.00 |
| Age | 17 | 4123 | 0.46 | 50.15 | 18.74 | 15.00 | 35.00 | 50.00 | 64.00 | 100.00 |
| Age | 18 | 4109 | 0.46 | 50.37 | 18.88 | 15.00 | 35.00 | 51.00 | 64.00 | 101.00 |
| Age | 19 | 4218 | 0.45 | 50.30 | 18.69 | 15.00 | 35.00 | 51.00 | 64.00 | 99.00 |
| Age | 20 | 4340 | 0.43 | 50.58 | 18.64 | 15.00 | 35.00 | 51.00 | 64.00 | 99.00 |
| Age | 21 | 4453 | 0.42 | 51.10 | 18.60 | 15.00 | 36.00 | 52.00 | 65.00 | 98.00 |
| Gender | 1 | 4437 | 0.42 | 58 % (women) |  |  |  |  |  |  |
| Gender | 2 | 4955 | 0.35 | 59 % (women) |  |  |  |  |  |  |
| Gender | 3 | 4774 | 0.38 | 60 % (women) |  |  |  |  |  |  |
| Gender | 4 | 4817 | 0.37 | 60 % (women) |  |  |  |  |  |  |
| Gender | 5 | 4847 | 0.37 | 61 % (women) |  |  |  |  |  |  |
| Gender | 6 | 4950 | 0.35 | 61 % (women) |  |  |  |  |  |  |
| Gender | 7 | 5073 | 0.34 | 62 % (women) |  |  |  |  |  |  |
| Gender | 8 | 5008 | 0.35 | 61 % (women) |  |  |  |  |  |  |
| Gender | 9 | 4883 | 0.36 | 61 % (women) |  |  |  |  |  |  |
| Gender | 10 | 4002 | 0.48 | 60 % (women) |  |  |  |  |  |  |
| Gender | 11 | 4049 | 0.47 | 61 % (women) |  |  |  |  |  |  |
| Gender | 12 | 4091 | 0.47 | 61 % (women) |  |  |  |  |  |  |
| Gender | 13 | 4095 | 0.47 | 61 % (women) |  |  |  |  |  |  |
| Gender | 14 | 4130 | 0.46 | 61 % (women) |  |  |  |  |  |  |
| Gender | 15 | 3991 | 0.48 | 61 % (women) |  |  |  |  |  |  |
| Gender | 16 | 4080 | 0.47 | 62 % (women) |  |  |  |  |  |  |
| Gender | 17 | 4123 | 0.46 | 62 % (women) |  |  |  |  |  |  |
| Gender | 18 | 4109 | 0.46 | 62 % (women) |  |  |  |  |  |  |
| Gender | 19 | 4218 | 0.45 | 63 % (women) |  |  |  |  |  |  |
| Gender | 20 | 4340 | 0.43 | 63 % (women) |  |  |  |  |  |  |
| Gender | 21 | 4453 | 0.42 | 63 % (women) |  |  |  |  |  |  |
| **High PA** |  |  |  |  |  |  |  |  |  |  |
| IN_NLE | 1 | 14964 | 0.37 | 0.57 | 0.83 | 0.00 | 0.00 | 0.00 | 1.00 | 8.00 |
| IN_NLE | 2 | 16155 | 0.32 | 0.55 | 0.79 | 0.00 | 0.00 | 0.00 | 1.00 | 5.00 |
| IN_NLE | 3 | 15296 | 0.35 | 0.55 | 0.81 | 0.00 | 0.00 | 0.00 | 1.00 | 7.00 |
| IN_NLE | 4 | 15221 | 0.36 | 0.53 | 0.80 | 0.00 | 0.00 | 0.00 | 1.00 | 8.00 |
| IN_NLE | 5 | 14956 | 0.37 | 0.52 | 0.78 | 0.00 | 0.00 | 0.00 | 1.00 | 8.00 |
| IN_NLE | 6 | 15156 | 0.36 | 0.49 | 0.76 | 0.00 | 0.00 | 0.00 | 1.00 | 8.00 |
| IN_NLE | 7 | 15260 | 0.35 | 0.50 | 0.77 | 0.00 | 0.00 | 0.00 | 1.00 | 6.00 |
| IN_NLE | 8 | 14931 | 0.37 | 0.51 | 0.77 | 0.00 | 0.00 | 0.00 | 1.00 | 6.00 |
| IN_NLE | 9 | 14531 | 0.38 | 0.51 | 0.80 | 0.00 | 0.00 | 0.00 | 1.00 | 6.00 |
| IN_NLE | 10 | 12122 | 0.49 | 0.52 | 0.79 | 0.00 | 0.00 | 0.00 | 1.00 | 8.00 |
| IN_NLE | 11 | 12056 | 0.49 | 0.57 | 0.83 | 0.00 | 0.00 | 0.00 | 1.00 | 6.00 |
| IN_NLE | 12 | 12066 | 0.49 | 0.50 | 0.78 | 0.00 | 0.00 | 0.00 | 1.00 | 7.00 |
| IN_NLE | 13 | 11838 | 0.50 | 0.52 | 0.79 | 0.00 | 0.00 | 0.00 | 1.00 | 6.00 |
| IN_NLE | 14 | 11855 | 0.50 | 0.51 | 0.81 | 0.00 | 0.00 | 0.00 | 1.00 | 7.00 |
| IN_NLE | 15 | 11273 | 0.52 | 0.54 | 0.82 | 0.00 | 0.00 | 0.00 | 1.00 | 8.00 |
| IN_NLE | 16 | 11271 | 0.52 | 0.47 | 0.76 | 0.00 | 0.00 | 0.00 | 1.00 | 7.00 |
| IN_NLE | 17 | 11517 | 0.51 | 0.52 | 0.80 | 0.00 | 0.00 | 0.00 | 1.00 | 7.00 |
| IN_NLE | 18 | 11289 | 0.52 | 0.50 | 0.80 | 0.00 | 0.00 | 0.00 | 1.00 | 8.00 |
| IN_NLE | 19 | 11574 | 0.51 | 0.51 | 0.83 | 0.00 | 0.00 | 0.00 | 1.00 | 8.00 |
| IN_NLE | 20 | 11859 | 0.50 | 0.47 | 0.77 | 0.00 | 0.00 | 0.00 | 1.00 | 8.00 |
| IN_NLE | 21 | 12240 | 0.48 | 0.53 | 0.82 | 0.00 | 0.00 | 0.00 | 1.00 | 6.00 |
| DE_NLE | 1 | 14875 | 0.37 | 0.11 | 0.36 | 0.00 | 0.00 | 0.00 | 0.00 | 4.00 |
| DE_NLE | 2 | 16112 | 0.32 | 0.10 | 0.34 | 0.00 | 0.00 | 0.00 | 0.00 | 3.00 |
| DE_NLE | 3 | 15261 | 0.35 | 0.09 | 0.33 | 0.00 | 0.00 | 0.00 | 0.00 | 3.00 |
| DE_NLE | 4 | 15152 | 0.36 | 0.10 | 0.33 | 0.00 | 0.00 | 0.00 | 0.00 | 3.00 |
| DE_NLE | 5 | 14918 | 0.37 | 0.10 | 0.33 | 0.00 | 0.00 | 0.00 | 0.00 | 4.00 |
| DE_NLE | 6 | 15102 | 0.36 | 0.09 | 0.32 | 0.00 | 0.00 | 0.00 | 0.00 | 4.00 |
| DE_NLE | 7 | 15205 | 0.36 | 0.10 | 0.33 | 0.00 | 0.00 | 0.00 | 0.00 | 3.00 |
| DE_NLE | 8 | 14854 | 0.37 | 0.11 | 0.36 | 0.00 | 0.00 | 0.00 | 0.00 | 3.00 |
| DE_NLE | 9 | 14459 | 0.39 | 0.09 | 0.33 | 0.00 | 0.00 | 0.00 | 0.00 | 4.00 |
| DE_NLE | 10 | 12028 | 0.49 | 0.10 | 0.34 | 0.00 | 0.00 | 0.00 | 0.00 | 4.00 |
| DE_NLE | 11 | 11982 | 0.49 | 0.10 | 0.33 | 0.00 | 0.00 | 0.00 | 0.00 | 3.00 |
| DE_NLE | 12 | 11963 | 0.49 | 0.10 | 0.34 | 0.00 | 0.00 | 0.00 | 0.00 | 4.00 |
| DE_NLE | 13 | 11747 | 0.50 | 0.10 | 0.33 | 0.00 | 0.00 | 0.00 | 0.00 | 3.00 |
| DE_NLE | 14 | 11808 | 0.50 | 0.10 | 0.34 | 0.00 | 0.00 | 0.00 | 0.00 | 4.00 |
| DE_NLE | 15 | 11171 | 0.53 | 0.10 | 0.34 | 0.00 | 0.00 | 0.00 | 0.00 | 4.00 |
| DE_NLE | 16 | 11182 | 0.53 | 0.08 | 0.31 | 0.00 | 0.00 | 0.00 | 0.00 | 3.00 |
| DE_NLE | 17 | 11429 | 0.52 | 0.09 | 0.32 | 0.00 | 0.00 | 0.00 | 0.00 | 4.00 |
| DE_NLE | 18 | 11230 | 0.52 | 0.09 | 0.32 | 0.00 | 0.00 | 0.00 | 0.00 | 4.00 |
| DE_NLE | 19 | 11571 | 0.51 | 0.11 | 0.36 | 0.00 | 0.00 | 0.00 | 0.00 | 4.00 |
| DE_NLE | 20 | 11819 | 0.50 | 0.09 | 0.32 | 0.00 | 0.00 | 0.00 | 0.00 | 4.00 |
| DE_NLE | 21 | 12206 | 0.48 | 0.08 | 0.30 | 0.00 | 0.00 | 0.00 | 0.00 | 3.00 |
| LIFE | 1 | 14719 | 0.38 | 7.99 | 1.46 | 0.00 | 7.00 | 8.00 | 9.00 | 10.00 |
| LIFE | 2 | 16001 | 0.32 | 8.09 | 1.39 | 0.00 | 7.00 | 8.00 | 9.00 | 10.00 |
| LIFE | 3 | 15118 | 0.36 | 8.04 | 1.42 | 0.00 | 7.00 | 8.00 | 9.00 | 10.00 |
| LIFE | 4 | 14998 | 0.36 | 8.01 | 1.36 | 0.00 | 7.00 | 8.00 | 9.00 | 10.00 |
| LIFE | 5 | 14747 | 0.38 | 7.99 | 1.36 | 0.00 | 7.00 | 8.00 | 9.00 | 10.00 |
| LIFE | 6 | 14949 | 0.37 | 8.02 | 1.33 | 0.00 | 7.00 | 8.00 | 9.00 | 10.00 |
| LIFE | 7 | 15024 | 0.36 | 8.00 | 1.30 | 0.00 | 7.00 | 8.00 | 9.00 | 10.00 |
| LIFE | 8 | 14713 | 0.38 | 8.00 | 1.31 | 0.00 | 7.00 | 8.00 | 9.00 | 10.00 |
| LIFE | 9 | 14346 | 0.39 | 7.99 | 1.33 | 0.00 | 7.00 | 8.00 | 9.00 | 10.00 |
| LIFE | 10 | 11921 | 0.50 | 8.05 | 1.34 | 0.00 | 7.00 | 8.00 | 9.00 | 10.00 |
| LIFE | 11 | 11846 | 0.50 | 8.03 | 1.34 | 0.00 | 7.00 | 8.00 | 9.00 | 10.00 |
| LIFE | 12 | 11831 | 0.50 | 8.02 | 1.33 | 0.00 | 7.00 | 8.00 | 9.00 | 10.00 |
| LIFE | 13 | 11591 | 0.51 | 8.02 | 1.34 | 0.00 | 7.00 | 8.00 | 9.00 | 10.00 |
| LIFE | 14 | 11641 | 0.51 | 8.05 | 1.34 | 0.00 | 7.00 | 8.00 | 9.00 | 10.00 |
| LIFE | 15 | 11037 | 0.53 | 8.03 | 1.34 | 0.00 | 7.00 | 8.00 | 9.00 | 10.00 |
| LIFE | 16 | 11072 | 0.53 | 8.01 | 1.33 | 0.00 | 7.00 | 8.00 | 9.00 | 10.00 |
| LIFE | 17 | 11276 | 0.52 | 8.05 | 1.34 | 0.00 | 7.00 | 8.00 | 9.00 | 10.00 |
| LIFE | 18 | 11095 | 0.53 | 8.06 | 1.34 | 0.00 | 7.00 | 8.00 | 9.00 | 10.00 |
| LIFE | 19 | 11398 | 0.52 | 8.06 | 1.31 | 0.00 | 7.00 | 8.00 | 9.00 | 10.00 |
| LIFE | 20 | 11651 | 0.51 | 8.04 | 1.32 | 0.00 | 7.00 | 8.00 | 9.00 | 10.00 |
| LIFE | 21 | 12021 | 0.49 | 8.06 | 1.29 | 0.00 | 7.00 | 8.00 | 9.00 | 10.00 |
| Age | 1 | 14717 | 0.38 | 42.42 | 17.04 | 15.00 | 29.00 | 41.00 | 54.00 | 97.00 |
| Age | 2 | 16000 | 0.32 | 44.19 | 16.88 | 16.00 | 31.00 | 43.00 | 56.00 | 98.00 |
| Age | 3 | 15112 | 0.36 | 42.90 | 17.45 | 15.00 | 29.00 | 42.00 | 56.00 | 99.00 |
| Age | 4 | 14996 | 0.36 | 43.00 | 17.75 | 15.00 | 28.00 | 42.00 | 56.00 | 100.00 |
| Age | 5 | 14745 | 0.38 | 42.96 | 18.01 | 15.00 | 28.00 | 42.00 | 56.00 | 94.00 |
| Age | 6 | 14948 | 0.37 | 43.28 | 18.15 | 15.00 | 28.00 | 43.00 | 57.00 | 94.00 |
| Age | 7 | 15012 | 0.36 | 43.46 | 18.37 | 15.00 | 27.00 | 43.00 | 57.00 | 93.00 |
| Age | 8 | 14706 | 0.38 | 43.38 | 18.35 | 15.00 | 27.00 | 43.00 | 57.00 | 93.00 |
| Age | 9 | 14344 | 0.39 | 43.36 | 18.54 | 15.00 | 27.00 | 42.00 | 57.00 | 95.00 |
| Age | 10 | 11917 | 0.50 | 43.68 | 18.45 | 15.00 | 27.00 | 43.00 | 58.00 | 96.00 |
| Age | 11 | 11841 | 0.50 | 43.88 | 18.58 | 15.00 | 27.50 | 43.00 | 58.00 | 97.00 |
| Age | 12 | 11827 | 0.50 | 43.86 | 18.64 | 15.00 | 27.00 | 43.00 | 58.00 | 96.00 |
| Age | 13 | 11589 | 0.51 | 43.98 | 18.79 | 15.00 | 27.00 | 43.00 | 58.00 | 98.00 |
| Age | 14 | 11638 | 0.51 | 44.46 | 18.93 | 15.00 | 28.00 | 43.00 | 59.00 | 99.00 |
| Age | 15 | 11036 | 0.53 | 44.26 | 18.93 | 15.00 | 28.00 | 43.00 | 59.00 | 99.00 |
| Age | 16 | 11065 | 0.53 | 44.71 | 18.99 | 15.00 | 28.00 | 43.00 | 60.00 | 100.00 |
| Age | 17 | 11270 | 0.52 | 45.13 | 19.06 | 15.00 | 29.00 | 43.50 | 60.00 | 97.00 |
| Age | 18 | 11088 | 0.53 | 45.24 | 19.19 | 15.00 | 29.00 | 43.00 | 60.00 | 98.00 |
| Age | 19 | 11393 | 0.52 | 45.59 | 19.15 | 15.00 | 29.00 | 44.00 | 61.00 | 99.00 |
| Age | 20 | 11645 | 0.51 | 45.88 | 19.28 | 15.00 | 30.00 | 44.00 | 61.00 | 100.00 |
| Age | 21 | 12016 | 0.49 | 46.47 | 19.44 | 15.00 | 30.00 | 44.00 | 62.00 | 101.00 |
| Gender | 1 | 14717 | 0.38 | 51 % (women) |  |  |  |  |  |  |
| Gender | 2 | 16000 | 0.32 | 51 % (women) |  |  |  |  |  |  |
| Gender | 3 | 15112 | 0.36 | 51 % (women) |  |  |  |  |  |  |
| Gender | 4 | 14996 | 0.36 | 51 % (women) |  |  |  |  |  |  |
| Gender | 5 | 14745 | 0.38 | 51 % (women) |  |  |  |  |  |  |
| Gender | 6 | 14948 | 0.37 | 51 % (women) |  |  |  |  |  |  |
| Gender | 7 | 15012 | 0.36 | 51 % (women) |  |  |  |  |  |  |
| Gender | 8 | 14706 | 0.38 | 51 % (women) |  |  |  |  |  |  |
| Gender | 9 | 14344 | 0.39 | 51 % (women) |  |  |  |  |  |  |
| Gender | 10 | 11917 | 0.50 | 51 % (women) |  |  |  |  |  |  |
| Gender | 11 | 11841 | 0.50 | 51 % (women) |  |  |  |  |  |  |
| Gender | 12 | 11827 | 0.50 | 51 % (women) |  |  |  |  |  |  |
| Gender | 13 | 11589 | 0.51 | 51 % (women) |  |  |  |  |  |  |
| Gender | 14 | 11638 | 0.51 | 51 % (women) |  |  |  |  |  |  |
| Gender | 15 | 11036 | 0.53 | 51 % (women) |  |  |  |  |  |  |
| Gender | 16 | 11065 | 0.53 | 51 % (women) |  |  |  |  |  |  |
| Gender | 17 | 11270 | 0.52 | 51 % (women) |  |  |  |  |  |  |
| Gender | 18 | 11088 | 0.53 | 51 % (women) |  |  |  |  |  |  |
| Gender | 19 | 11393 | 0.52 | 51 % (women) |  |  |  |  |  |  |
| Gender | 20 | 11645 | 0.51 | 51 % (women) |  |  |  |  |  |  |
| Gender | 21 | 12016 | 0.49 | 51 % (women) |  |  |  |  |  |  |
| *Note.* IN_NLE = independent negative life events; DE_NLE = dependent negative life events; LIFE = life satisfaction. | | | | | | | | | | |

| **Table 2**  *Parameters and Model Fit Indices for the Multiple-Group Random Intercept Cross-Lagged Panel Model – HILDA Data* | | | | |
| --- | --- | --- | --- | --- |
| low PA | RI-CLPM | | |  |
|  | Est. | *SE* | *p* | Stand. Est […] |
| $\gamma_{IN\_NLE\sim IN\_NLE}$ | 0.166 | 0.007 | <.001 | [0.154, 0.183] |
| $\beta_{IN\_NLE\sim DE\_NLE}$ | –0.003 | 0.013 | .791 | [–0.001, –0.002] |
| $\beta_{IN\_NLE\sim LIFE}$ | –0.007 | 0.004 | .054 | [–0.010, –0.012] |
| $\gamma_{DE\_NLE\sim DE\_NLE}$ | 0.189 | 0.009 | <.001 | [0.163, 0.227] |
| $\beta_{DE\_NLE\sim IN\_NLE}$ | 0.000 | 0.002 | .961 | 0 |
| $\beta_{DE\_NLE\sim LIFE}$ | –0.008 | 0.002 | <.001 | [–0.027, –0.037] |
| $\gamma_{LIFE\sim LIFE}$ | 0.251 | 0.008 | <.001 | [0.235, 0.273] |
| $\beta_{LIFE\sim DE\_NLE}$ | –0.093 | 0.021 | <.001 | [–0.024, –0.030] |
| $\beta_{LIFE\sim IN\_NLE}$ | –0.015 | 0.008 | .053 | [–0.009, –0.011] |
| $T_{IN\_NLE\sim\sim DE\_NLE}$ | 0.021 | 0.002 | <.001 | 0.384 |
| $T_{IN\_NLE\sim\sim LIFE}$ | –0.124 | 0.012 | <.001 | –0.258 |
| $T_{DE\_NLE\sim\sim LIFE}$ | –0.069 | 0.004 | <.001 | –0.415 |
|  |  |  |  |  |
| high PA |  |  |  |  |
|  | Est. | *SE* | *p* | Stand. Est […] |
| $\gamma_{IN\_NLE\sim IN\_NLE}$ | 0.153 | 0.004 | <.001 | [0.145, 0.165] |
| $\beta_{IN\_NLE\sim DE\_NLE}$ | 0.012 | 0.007 | .099 | [0.005, 0.006] |
| $\beta_{IN\_NLE\sim LIFE}$ | –0.013 | 0.002 | <.001 | [–0.017, –0.022] |
| $\gamma_{DE\_NLE\sim DE\_NLE}$ | 0.152 | 0.005 | <.001 | [0.135, 0.175] |
| $\beta_{DE\_NLE\sim IN\_NLE}$ | 0.002 | 0.001 | .187 | 0.004 |
| $\beta_{DE\_NLE\sim LIFE}$ | –0.015 | 0.001 | <.001 | [–0.042, –0.055] |
| $\gamma_{LIFE\sim LIFE}$ | 0.261 | 0.005 | <.001 | [0.256, 0.279] |
| $\beta_{LIFE\sim DE\_NLE}$ | –0.096 | 0.010 | <.001 | [–0.029, –0.033] |
| $\beta_{LIFE\sim IN\_NLE}$ | –0.015 | 0.004 | <.001 | [–0.010, –0.011] |
| $T_{IN\_NLE\sim\sim DE\_NLE}$ | 0.018 | 0.001 | <.001 | 0.418 |
| $T_{IN\_NLE\sim\sim LIFE}$ | –0.063 | 0.005 | <.001 | –0.186 |
| $T_{DE\_NLE\sim\sim LIFE}$ | –0.045 | 0.002 | <.001 | –0.385 |
|  |  |  |  |  |
| AIC | 1790114.868 |  |  |  |
| BIC | 1794574.109 |  |  |  |
| Robust CFI | .956 |  |  |  |
| Robust TLI | .954 |  |  |  |
| Robust RMSEA [90% CI] | .022 [.021;.022] |  |  |  |
| SRMR | .031 |  |  |  |
| Degrees of freedom | 3754 |  |  |  |
| Number of parameters | 880 | |  |  |
| *Note.* $\gamma$ = autoregressive effects; $\beta$ = cross-lagged effects;$T$ = random intercept factors; IN_NLE = independent negative life events; DE_NLE = dependent negative life events; LIFE = life satisfaction; PA = moderate-to-vigorous physical activity; [ ] = describes the values between which the standardized effects vary. | | | | |

| **Table 3**  *Descriptive Statistics for Mean Age, Gender, and the Distributions of Independent Negative Life Events, Dependent Negative Life Events, and Life Satisfaction across all Time Points and by Group* *– SOEP Data* | | | | | | | | | | |
| --- | --- | --- | --- | --- | --- | --- | --- | --- | --- | --- |
| Variable | Wave | n_Missing | Complete Rate | *M* | *SD* | 0th Percentile | 25th Percentile | 50th Percentile | 75th Percentile | 100th Percentile |
| **Low PA** |  |  |  |  |  |  |  |  |  |  |
| IN_NLE | 1 | 0 | 1.00 | 0.03 | 0.16 | 0.00 | 0.00 | 0.00 | 0.00 | 2.00 |
| IN_NLE | 2 | 0 | 1.00 | 0.03 | 0.17 | 0.00 | 0.00 | 0.00 | 0.00 | 2.00 |
| IN_NLE | 3 | 0 | 1.00 | 0.03 | 0.16 | 0.00 | 0.00 | 0.00 | 0.00 | 2.00 |
| IN_NLE | 4 | 0 | 1.00 | 0.02 | 0.15 | 0.00 | 0.00 | 0.00 | 0.00 | 2.00 |
| IN_NLE | 5 | 0 | 1.00 | 0.03 | 0.16 | 0.00 | 0.00 | 0.00 | 0.00 | 2.00 |
| IN_NLE | 6 | 0 | 1.00 | 0.03 | 0.16 | 0.00 | 0.00 | 0.00 | 0.00 | 2.00 |
| IN_NLE | 7 | 0 | 1.00 | 0.03 | 0.17 | 0.00 | 0.00 | 0.00 | 0.00 | 2.00 |
| IN_NLE | 8 | 0 | 1.00 | 0.04 | 0.20 | 0.00 | 0.00 | 0.00 | 0.00 | 4.00 |
| DE_NLE | 1 | 0 | 1.00 | 0.09 | 0.30 | 0.00 | 0.00 | 0.00 | 0.00 | 3.00 |
| DE_NLE | 2 | 1 | 1.00 | 0.08 | 0.28 | 0.00 | 0.00 | 0.00 | 0.00 | 3.00 |
| DE_NLE | 3 | 4 | 1.00 | 0.09 | 0.30 | 0.00 | 0.00 | 0.00 | 0.00 | 3.00 |
| DE_NLE | 4 | 0 | 1.00 | 0.10 | 0.31 | 0.00 | 0.00 | 0.00 | 0.00 | 3.00 |
| DE_NLE | 5 | 13 | 1.00 | 0.10 | 0.31 | 0.00 | 0.00 | 0.00 | 0.00 | 3.00 |
| DE_NLE | 6 | 2966 | 0.91 | 0.12 | 0.33 | 0.00 | 0.00 | 0.00 | 0.00 | 3.00 |
| DE_NLE | 7 | 313 | 0.99 | 0.12 | 0.33 | 0.00 | 0.00 | 0.00 | 0.00 | 3.00 |
| DE_NLE | 8 | 378 | 0.98 | 0.09 | 0.29 | 0.00 | 0.00 | 0.00 | 0.00 | 3.00 |
| LIFE | 1 | 52 | 1.00 | 6.95 | 1.78 | 0.00 | 6.00 | 7.00 | 8.00 | 10.00 |
| LIFE | 2 | 59 | 1.00 | 6.98 | 1.78 | 0.00 | 6.00 | 7.00 | 8.00 | 10.00 |
| LIFE | 3 | 2602 | 0.91 | 7.18 | 1.74 | 0.00 | 6.00 | 8.00 | 8.00 | 10.00 |
| LIFE | 4 | 72 | 1.00 | 7.31 | 1.75 | 0.00 | 7.00 | 8.00 | 8.00 | 10.00 |
| LIFE | 5 | 63 | 1.00 | 7.38 | 1.73 | 0.00 | 7.00 | 8.00 | 8.00 | 10.00 |
| LIFE | 6 | 65 | 1.00 | 7.34 | 1.77 | 0.00 | 7.00 | 8.00 | 8.00 | 10.00 |
| LIFE | 7 | 82 | 1.00 | 7.54 | 1.71 | 0.00 | 7.00 | 8.00 | 9.00 | 10.00 |
| LIFE | 8 | 101 | 1.00 | 7.51 | 1.72 | 0.00 | 7.00 | 8.00 | 9.00 | 10.00 |
| Age | 1 | 0 | 1.00 | 48.98 | 17.52 | 18.00 | 36.00 | 48.00 | 63.00 | 98.00 |
| Age | 2 | 0 | 1.00 | 49.91 | 17.71 | 17.00 | 37.00 | 50.00 | 64.00 | 100.00 |
| Age | 3 | 0 | 1.00 | 47.70 | 16.94 | 16.00 | 35.00 | 46.00 | 60.00 | 101.00 |
| Age | 4 | 0 | 1.00 | 46.97 | 17.06 | 16.00 | 34.00 | 45.00 | 59.00 | 103.00 |
| Age | 5 | 0 | 1.00 | 47.75 | 17.48 | 17.00 | 34.00 | 47.00 | 60.00 | 105.00 |
| Age | 6 | 0 | 1.00 | 45.97 | 17.49 | 16.00 | 32.00 | 45.00 | 58.00 | 102.00 |
| Age | 7 | 0 | 1.00 | 48.14 | 17.38 | 15.00 | 35.00 | 48.00 | 60.00 | 102.00 |
| Age | 8 | 0 | 1.00 | 48.23 | 17.40 | 18.00 | 34.00 | 48.00 | 61.00 | 102.00 |
| Gender | 1 | 0 | 1.00 | 52 % (women) |  |  |  |  |  |  |
| Gender | 2 | 0 | 1.00 | 52 % (women) |  |  |  |  |  |  |
| Gender | 3 | 0 | 1.00 | 55 % (women) |  |  |  |  |  |  |
| Gender | 4 | 0 | 1.00 | 54 % (women) |  |  |  |  |  |  |
| Gender | 5 | 0 | 1.00 | 54 % (women) |  |  |  |  |  |  |
| Gender | 6 | 0 | 1.00 | 51 % (women) |  |  |  |  |  |  |
| Gender | 7 | 7 | 1.00 | 51 % (women) |  |  |  |  |  |  |
| Gender | 8 | 8 | 1.00 | 51 % (women) |  |  |  |  |  |  |
| **High PA** |  |  |  |  |  |  |  |  |  |  |
| IN_NLE | 1 | 0 | 1.00 | 0.03 | 0.16 | 0.00 | 0.00 | 0.00 | 0.00 | 2.00 |
| IN_NLE | 2 | 0 | 1.00 | 0.03 | 0.16 | 0.00 | 0.00 | 0.00 | 0.00 | 1.00 |
| IN_NLE | 3 | 0 | 1.00 | 0.02 | 0.16 | 0.00 | 0.00 | 0.00 | 0.00 | 2.00 |
| IN_NLE | 4 | 0 | 1.00 | 0.02 | 0.15 | 0.00 | 0.00 | 0.00 | 0.00 | 2.00 |
| IN_NLE | 5 | 0 | 1.00 | 0.03 | 0.16 | 0.00 | 0.00 | 0.00 | 0.00 | 2.00 |
| IN_NLE | 6 | 0 | 1.00 | 0.03 | 0.16 | 0.00 | 0.00 | 0.00 | 0.00 | 2.00 |
| IN_NLE | 7 | 0 | 1.00 | 0.03 | 0.18 | 0.00 | 0.00 | 0.00 | 0.00 | 2.00 |
| IN_NLE | 8 | 0 | 1.00 | 0.04 | 0.19 | 0.00 | 0.00 | 0.00 | 0.00 | 2.00 |
| DE_NLE | 1 | 0 | 1.00 | 0.06 | 0.25 | 0.00 | 0.00 | 0.00 | 0.00 | 3.00 |
| DE_NLE | 2 | 0 | 1.00 | 0.05 | 0.22 | 0.00 | 0.00 | 0.00 | 0.00 | 3.00 |
| DE_NLE | 3 | 0 | 1.00 | 0.05 | 0.23 | 0.00 | 0.00 | 0.00 | 0.00 | 3.00 |
| DE_NLE | 4 | 0 | 1.00 | 0.06 | 0.25 | 0.00 | 0.00 | 0.00 | 0.00 | 2.00 |
| DE_NLE | 5 | 5 | 1.00 | 0.06 | 0.25 | 0.00 | 0.00 | 0.00 | 0.00 | 3.00 |
| DE_NLE | 6 | 785 | 0.94 | 0.07 | 0.27 | 0.00 | 0.00 | 0.00 | 0.00 | 2.00 |
| DE_NLE | 7 | 7 | 1.00 | 0.07 | 0.27 | 0.00 | 0.00 | 0.00 | 0.00 | 3.00 |
| DE_NLE | 8 | 43 | 1.00 | 0.06 | 0.24 | 0.00 | 0.00 | 0.00 | 0.00 | 2.00 |
| LIFE | 1 | 11 | 1.00 | 7.42 | 1.50 | 0.00 | 7.00 | 8.00 | 8.00 | 10.00 |
| LIFE | 2 | 18 | 1.00 | 7.40 | 1.55 | 0.00 | 7.00 | 8.00 | 8.00 | 10.00 |
| LIFE | 3 | 948 | 0.91 | 7.58 | 1.47 | 0.00 | 7.00 | 8.00 | 9.00 | 10.00 |
| LIFE | 4 | 21 | 1.00 | 7.66 | 1.47 | 0.00 | 7.00 | 8.00 | 9.00 | 10.00 |
| LIFE | 5 | 28 | 1.00 | 7.67 | 1.51 | 0.00 | 7.00 | 8.00 | 9.00 | 10.00 |
| LIFE | 6 | 22 | 1.00 | 7.60 | 1.58 | 0.00 | 7.00 | 8.00 | 9.00 | 10.00 |
| LIFE | 7 | 31 | 1.00 | 7.82 | 1.50 | 0.00 | 7.00 | 8.00 | 9.00 | 10.00 |
| LIFE | 8 | 37 | 1.00 | 7.71 | 1.53 | 0.00 | 7.00 | 8.00 | 9.00 | 10.00 |
| Age | 1 | 0 | 1.00 | 45.38 | 15.93 | 18.00 | 33.00 | 45.00 | 58.00 | 93.00 |
| Age | 2 | 0 | 1.00 | 46.69 | 16.52 | 17.00 | 33.00 | 47.00 | 60.00 | 94.00 |
| Age | 3 | 0 | 1.00 | 45.62 | 15.80 | 16.00 | 34.00 | 45.00 | 57.00 | 96.00 |
| Age | 4 | 0 | 1.00 | 45.77 | 16.70 | 16.00 | 33.00 | 45.00 | 58.00 | 98.00 |
| Age | 5 | 0 | 1.00 | 46.11 | 17.30 | 17.00 | 32.00 | 46.00 | 59.00 | 100.00 |
| Age | 6 | 0 | 1.00 | 44.97 | 17.72 | 16.00 | 29.00 | 45.00 | 58.00 | 102.00 |
| Age | 7 | 0 | 1.00 | 47.68 | 17.58 | 18.00 | 33.00 | 49.00 | 60.00 | 98.00 |
| Age | 8 | 0 | 1.00 | 47.64 | 17.88 | 18.00 | 32.00 | 49.00 | 61.00 | 100.00 |
| Gender | 1 | 0 | 1.00 | 55 % (women) |  |  |  |  |  |  |
| Gender | 2 | 0 | 1.00 | 55 % (women) |  |  |  |  |  |  |
| Gender | 3 | 0 | 1.00 | 56 % (women) |  |  |  |  |  |  |
| Gender | 4 | 0 | 1.00 | 56 % (women) |  |  |  |  |  |  |
| Gender | 5 | 0 | 1.00 | 55 % (women) |  |  |  |  |  |  |
| Gender | 6 | 0 | 1.00 | 51 % (women) |  |  |  |  |  |  |
| Gender | 7 | 3 | 1.00 | 51 % (women) |  |  |  |  |  |  |
| Gender | 8 | 3 | 1.00 | 52 % (women) |  |  |  |  |  |  |
| *Note.* IN_NLE = independent negative life events; DE_NLE = dependent negative life events; LIFE = life satisfaction. | | | | | | | | | | |

| **Table 4**  *Parameters and Model Fit Indices for the Multiple-Group Random Intercept Cross-Lagged Panel Model – SOEP Data* |
| --- |

| low PA | RI-CLPM | | |  |
| --- | --- | --- | --- | --- |
|  | Est. | *SE* | *p* | Stand. Est […] |
| $\gamma_{IN\_NLE\sim IN\_NLE}$ | 0.013 | 0.004 | .002 | [0.010, 0.014] |
| $\beta_{IN\_NLE\sim DE\_NLE}$ | 0.001 | 0.003 | .824 | 0.001 |
| $\beta_{IN\_NLE\sim LIFE}$ | 0.000 | 0.001 | .590 | –0.002 |
| $\gamma_{DE\_NLE\sim DE\_NLE}$ | 0.184 | 0.011 | <.001 | [0.165, 0.208] |
| $\beta_{DE\_NLE\sim IN\_NLE}$ | –0.003 | 0.007 | .609 | –0.002 |
| $\beta_{DE\_NLE\sim LIFE}$ | –0.004 | 0.001 | <.001 | [–0.020, –0.024] |
| $\gamma_{LIFE\sim LIFE}$ | 0.158 | 0.008 | <.001 | [0.153, 0.163] |
| $\beta_{LIFE\sim DE\_NLE}$ | –0.104 | 0.029 | <.001 | [–0.019, –0.022] |
| $\beta_{LIFE\sim IN\_NLE}$ | –0.016 | 0.035 | .640 | –0.002 |
| $T_{IN\_NLE\sim\sim DE\_NLE}$ | 0.000 | 0.000 | .134 | –0.081 |
| $T_{IN\_NLE\sim\sim LIFE}$ | –0.004 | 0.001 | <.001 | –0.229 |
| $T_{DE\_NLE\sim\sim LIFE}$ | –0.057 | 0.003 | <.001 | –0.224 |
|  |  |  |  |  |
| high PA |  |  |  |  |
|  | Est. | *SE* | *p* | Stand. Est […] |
| $\gamma_{IN\_NLE\sim IN\_NLE}$ | 0.006 | 0.005 | .267 | [0.005, 0.006] |
| $\beta_{INLE\sim DE\_NLE}$ | 0.002 | 0.004 | .684 | 0.002 |
| $\beta_{IN\_NLE\sim LIFE}$ | –0.003 | 0.001 | .002 | [–0.015, –0.019] |
| $\gamma_{DE\_NLE\sim DE\_NLE}$ | 0.115 | 0.015 | <.001 | [0.107, 0.132] |
| $\beta_{DE\_NLE\sim IN\_NLE}$ | –0.019 | 0.007 | .003 | [–0.013, –0.016] |
| $\beta_{DE\_NLE\sim LIFE}$ | –0.007 | 0.002 | <.001 | [–0.033, –0.041] |
| $\gamma_{LIFE\sim LIFE}$ | 0.165 | 0.010 | <.001 | [0.155, 0.175] |
| $\beta_{LIFE\sim DE\_NLE}$ | –0.039 | 0.039 | .309 | [–0.007, –0.008] |
| $\beta_{LIFE\sim IN\_NLE}$ | 0.008 | 0.034 | .821 | 0.001 |
| $T_{IN\_NLE\sim\sim DE\_NLE}$ | 0.000 | 0.000 | .542 | –0.057 |
| $T_{IN\_NLE\sim\sim LIFE}$ | –0.001 | 0.001 | .230 | –0.085 |
| $T_{DE\_NLE\sim\sim LIFE}$ | –0.028 | 0.002 | <.001 | –0.230 |
|  |  |  |  |  |
| AIC | 829872.993 |  |  |  |
| BIC | 831923.116 |  |  |  |
| Robust CFI | .973 |  |  |  |
| Robust TLI | .969 |  |  |  |
| Robust RMSEA [90% CI] | .024 [.023;.026] |  |  |  |
| SRMR | .030 |  |  |  |
| Degrees of freedom | 475 |  |  |  |
| Number of parameters | 334 | |  |  |
| *Note.* $\gamma$ = autoregressive effects; $\beta$ = cross-lagged effects;$T$ = random intercept factors; IN_NLE = independent negative life events; DE_NLE = dependent negative life events; LIFE = life satisfaction; PA = sport participation in leisure time; [ ] = describes the values between which the standardized effects vary. | | | | |

**References**

Enders, C. K. (2008). A note on the use of missing auxiliary variables in full information maximum likelihood-based structural equation models. *Structural Equation Modeling: A Multidisciplinary Journal, 15*, 434–448. https://doi.org/10.1080/10705510802154307

Enders, C. K. (2010). *Applied missing data analysis.* Guilford Press.

Graham, J. W. (2003). Adding missing-data-relevant variables to FIML-based structural equation models. *Structural Equation Modeling: A Multidisciplinary Journal, 10*, 80–100. https://doi.org/10.1207/S15328007SEM1001_4

Hamaker, E. L., Kuiper, R. M., & Grasman, R. P. (2015). A critique of the cross‐lagged panel model. *Psychological Methods, 20*, 102–116. https://doi.org/10.1037/a0038889

Mulder, J. D., & Hamaker, E. L. (2021). Three extensions of the random intercept cross-lagged panel model. *Structural Equation Modeling: A Multidisciplinary Journal, 28*, 638–648. https://doi.org/10.1080/10705511.2020.1784738

Mund, M., & Nestler, S. (2019). Beyond the cross-lagged panel model: Next-generation tools for analyzing interdependencies across the life course. *Advances in Life Course Research, 41*, 100249. https://doi.org/10.1016/j.alcr.2018.10.002

Preacher, K. J., & Yaremych, H. E. (2023). Model selection in structural equation modeling. In R. H. Hoyle (Ed.) *Handbook of Structural Equation Modeling* (2nd Ed), pp. 206–222. Guilford.

Usami, S., Murayama, K., & Hamaker, E. L. (2019). A unified framework of longitudinal models to examine reciprocal relations. *Psychological Methods, 24*, 637–657. https://doi.org/10.1037/met0000210
